# Estimating genetic gains for tolerance to stress combinations in tropical maize hybrids

**DOI:** 10.3389/fgene.2022.1023318

**Published:** 2022-12-08

**Authors:** Abebe Menkir, Ibnou Dieng, Silvestro Meseka, Bunmi Bossey, Wende Mengesha, Oyekunle Muhyideen, Priscillia F. Riberio, Mmadou Coulibaly, Abdoul-Madjidou Yacoubou, Folusho A. Bankole, Gloria Boakyewaa Adu, Tayo Ojo

**Affiliations:** ^1^ International Institute of Tropical Agriculture (IITA), Ibadan, Nigeria; ^2^ Institute for Agricultural Research, Ahmadu Bello University, Zaria, Nigeria; ^3^ Crop Research Institute, Kumasi, Ghana; ^4^ Institute de Economic Rurale, Bamako, Mali; ^5^ National Institute of Agricultural Research of Benin/CRA, Cotonou, Benin; ^6^ University of Ilorin, Ilorin, Nigeria; ^7^ Savanna Agricultural Research Institute, Tamale, Ghana

**Keywords:** genetic gain, tolerance, stress combinations, maize, drought, hybrid

## Abstract

Maize is a strategic food crop in sub-Saharan Africa. However, most maize growing tropical savannas particularly in West and Central African experience the occurrence of frequent droughts and *Striga* infestation, resulting in 30–100% yield losses. This production zones need maize cultivars that combine tolerance to the two stresses. IITA in collaboration with national partners has thus employed a sequential selection scheme to incorporate both drought tolerance and *Striga* resistance in topical maize hybrids using reliable screening protocols. The main objective of the present study was therefore to use grain yield and other agronomic traits recorded in regional collaborative hybrid trials conducted for 8 years under manged stressful and non-stressful conditions and across rainfed field environments to estimate genetic gains in grain yields using mixed model analyses. The results showed significant (*p <* 0.05) annual yield gains of 11.89 kg ha^−1^ under manged drought stress (MDS) and 86.60 kg ha^−1^ under *Striga* infestation (STRIN) with concomitant yield increases of 62.65 kg ha^−1^ under full irrigation (WW), 102.44 kg ha^−1^ under *Striga* non-infested (STRNO) conditions and 53.11 kg ha^−1^ across rainfed field environments. Grain yield displayed significant but not strong genetic correlation of 0.41 ± 0.07 between MDS and STRIN, indicating that gene expression was not consistent across the two stress conditions. Furthermore, grain yield recorded in MET had significant moderate genetic correlations of 0.58 ± 0.06 and 0.44 ± 0.07It with MDS and STRIN, respectively. These results emphasize the need to screen inbred linens under both stress conditions to further enhance the rate of genetic gain in grain yield in hybrids for areas where the two stresses co-occur. Nonetheless, this study demonstrated that the sequential selection scheme has been successful in generating hybrids with dependable yields that can reduce chronic food deficits in rural communities experiencing simultaneous presence of drought and *S. hermonthica* infestation in their production fields.

## Introduction

Maize is a strategic food crop in sub-Saharan Africa (SSA) providing more than 30% of the dietary energy and feeding over 300 million people (Nuss and Tanumihardjo, 2010). The crop is currently produced on more than 43 million hectares in Africa with an annual production of over 90 million mt (FAO, 2022). The demand for maize as sources of food, feed and industrial use is increasing in SSA (Santpoort, 2020). The supply response to increasing demand for maize in SSA has been expanding its production into new areas. About 75% of crop production growth in Africa came from expansion of cultivated area and only 25% from improvements in grain yield (Jayne and Sanchez, 2021). On the other hand, smallholder farmers in SSA that cultivate maize primarily in rainfed cropping systems are facing shrinking farm sizes due to rapid rural population growth that limits further expansion of production into new areas (Jayne and Sanchez, 2021). As maize has also been a dominant crop particularly in the savannas where drought stress is common and a parasitic weed known as *Striga hermonthica* thrive (Ortiz, 2017), increasing maize production in existing farmlands to meet the increased demand faces a significant challenge. Drought can inflict yield losses of 30%–90% in maize depending on the severity and duration of the stress and crop growth stage (Sah et al., 2020), while severely *Striga* infested fields in Africa growing susceptible maize can sustain up to 100% yield loss (Kim et al., 2002; Ejeta, 2007). Moreover, the ongoing climate change induced increases in the intensity and frequency of drought and temperatures (Masih et al., 2014; Shiferaw et al., 2014; Tesfaye et al., 2015) coupled with anticipated enlargement of parasitic weed habitats will further diminish yields in tropical maize (Mohamed et al., 2007; Korres et al., 2016; Ronald, et al., 2017). Breeders working to support smallholder farmers in such target production environments should therefore develop maize varieties that can withstand the combined negative effects of drought and *Striga* to curtail yield losses and build resilience of farming communities.

As maize farmers in tropical savannas rarely confront a single type of stress in isolation, it becomes difficult for maize to deal with simultaneous occurrence of multiple stresses. When drought and *Striga* infestation co-occur in Smallholder farmers’ fields, they can inflict severe damage on crop growth and productivity leading to greater yield losses in comparison to the effect of each stress occurring separately (Mittler, 2006; Suzuki et al., 2014; Pandey et al., 2017). Appropriate and effective breeding strategies are thus required to develop maize varieties that deliver high yields under drought stress and *Striga* infestation as well as across diverse rainfed field growing environments. This is important because farmers primarily adopt stress tolerant crop varieties to manage the risks associated with cropping in areas affected by simultaneous presence of multiple stresses to minimize yield losses in their farms (Asfaw and Lipper, 2011; Wheeler and von Braun, 2013). Nonetheless, combining defence mechanisms conferring tolerance to drought and resistance to *Striga* in a single maize cultivar presents a significant challenge because these complex traits are regulated by many genes with small individual effects (Malenica et al., 2021).

In this regard, the International Institute of Tropical Agriculture (IITA) in collaboration with the national research partners implemented a breeding strategy to combine tolerance to drought with resistance to *Striga* in maize under the Drought Tolerant Maize for Africa (DTMA) and Stress Tolerant Maize for Africa (STMA) projects funded by Bill and Melinda Gates Foundation (Menkir et al., 2020). A sequential selection scheme was adopted to utilize the existing native genetic variation improved for tolerance only to one stress factor for developing promising maize hybrids with combined tolerance to drought stress and *Striga* infection. Limited understanding of genetic gains for combination of two or more stresses restricts the potential use of maize breeding to minimize economic losses (Malenica et al., 2021). Consequently, determining the effectiveness of the sequential selection scheme in achieving genetic gain for grain yield under stressful and non-stressful growing conditions is necessary to correct the breeding direction and develop new maize cultivars that deliver higher rates of productivity gains to smallholder farmers in areas affected by stress combinations.

Genetic gain has been estimated through evaluation of open-pollinated and hybrid maize varieties released over time in field trials conducted in common sets of environments (Duvick, 2005; Badu–Apraku et al., 2013a; Echarte et al., 2013; Omolara et al., 2014; Chen et al., 2016; Masuka et al., 2017a). Although such studies provide accurate genetic gain estimates by eliminating the confounding effects of differences in trial management practices and varying climatic conditions on genetic trends, finding genotypes representing each breeding cycle for organizing these trials and identifying testing environments that represent the target environments for which the genotypes were develop are often difficult (Rutkoski, 2019). In addition, these trials do not allow timely monitoring of the effectiveness of a breeding strategy to make the necessary adjustments. Multi-environment trials data that are regularly conducted in breeding programs with common checks included across years can estimate genetic gains (Eberhart, 1964). Sharma et al. (2012) determined genetic gains in a long-term wheat trial by regressing the difference in mean grain yield of the five highest yielding lines on the mean yield of a widely grown cultivar commonly used as a check and reported an annual yield gain of 0.68% over the common check cultivar. Crespo-Herrera et al. (2017) used a similar approach by applying a factor analytic model to estimate genetic gains from a historical data in wheat and found an annual yield gain of 1.67% relative to the widely cultivated check and 0.53% relative to the local check. Considering the dynamics of continually adding new genotypes and removal of inferior ones every year in breeding trials, many authors have recommended use of mixed models to analyse unbalanced multi-environment trial data and generate reliable estimates of genetic gains (Piepho and Mehring, 2006; de la Vega et al., 2007; Liu et al., 2015). Such analyses permit periodic reassessment of the effectiveness of existing selection schemes to change directions and develop better products encapsulating improvements in individual or combination of target traits.

IITA organizes regional cooperative trials every year to share elite hybrids formed from inbred lines developed through repeated selection first under artificial *Striga* infestation followed by selection under controlled drought stress. The elite hybrids are selected after undergoing three successive stages of performance evaluation under managed drought stress and full irrigation as well as under artificial *Striga* infested and non-infested conditions before entering the regional trials for extensive testing across locations. The long-term data sets recorded form these regional cooperative trials are therefore suitable to estimate genetic gains for grain yield under stressful and favourable growing conditions. Kumar et al. (2021) analysed multi-environment trial data sets for rice using a mixed model and reported annual yield gains of 0.68% under irrigation, 0.87 under moderate drought stress and 1.9% under severe drought stress conditions. The present study was therefore conducted to 1) examine yield gains under controlled drought stress and full irrigation, artificial *Striga* infested and non-infested conditions and across diverse rainfed field environments, 2) identify important traits contributing significantly to improvements in grain yield under stressful conditions, and 3) determine the usefulness of the sequential selection scheme in developing hybrids combining tolerance to draught with resistance to *S. hermonthica* that are also superior in agronomic performance across diverse rainfed field environments.

## Materials and methods

### Genetic materials

In the present study, we used data recorded from cooperative regional trials conducted under artificial *Striga* infestation and non-infested conditions, managed drought stress (MDS) and full irrigation (WW) as well as across many rainfed testing locations from 2012 to 2019. These regional trials consisted of 30–46 elite drought tolerant and *Striga* resistant (DTSTR) 3-way cross (H01-H112) hybrids, two *Striga* resistant (H113-H114) and 8 to 11 conventional commercial hybrids (H115-H142) plus a farmer preferred maize (H143) variety (Supplementary Table S1). The DTSTR 3-way cross hybrids were formed from inbred lines that had been subjected to eight generations (S8) of inbreeding with sequential selection first under artificial *Striga* infestation at Abuja and Mokwa followed by selection under managed drought stress conditions at Ikenne (Menkir et al., 2020). The commercial hybrids comprised two *Striga* resistant 3-way cross hybrids (STRCOM) received from Premier Seeds Nigeria Ltd as well as conventional top-cross, single-cross and three 3-way cross hybrids that were not specifically bred for resistance to *Striga* and tolerance to drought (CONCOM) supplied by national, regional, and multi-national seed companies and marketed in many African countries. The local maize variety (LOCAL) was a farmer preferred recycled hybrid or an improved open-pollinated maize variety commonly grown around the testing site where the regional trials were conducted. Out of the 112 DTSTR hybrids included in the regional trials, 42 were tested for 1 year, 26 for 2 years, 15 for 3 years, 12 for 4 years, six for 5 years, three for 6 years, five for 7 years and three for 8 years. Amongst the 30 commercial hybrids, 12 were tested for 1 year, 8 for 2 years, 2 for 3 years, 3 for 4 years, 2 for 5 years and one each for 6, 7 and 8 years (https://doi.org/10.25502/4xv7-bc93/d).

### Performance testing under manged drought stress and full irrigation

All regional hybrid trials were arranged in alpha lattice designs with three replications under managed drought stress (MDS) and full irrigation (WW) at the IITA experiment station in Ikenne (6^0^53′ N, 3^0^42’ E, altitude of 60 m) in Nigeria during the 2011/2012 to 2018/2019 dry seasons. In brief, drought stress was induced in a block by withdrawing irrigation from 35 days after planting to harvesting time of the trials, whereas an adjacent block received full irrigation every week through a sprinkler irrigation system from planting until the hybrids attained physiological maturity. Withdrawing irrigation 15–20 days before anthesis guarantees the presence of drought stresses coinciding with the most sensitive stages of maize (Bänziger et al., 2000). The amount of rainfall from the date of planting in November to end of March was not appreciable for seven of the 8 years at this station (Supplementary Table S2), leaving the maize crop planted during this period to rely mainly on irrigation. Each hybrid was planted in two 4 m long rows spaced 0.75 m apart with 0.25 m spacing between plants within a row. Two seeds were planted in each hill and one plant was removed after emergence to attain a population density of 53,333 plants per ha. A compound fertilizer of 60 kg N, 60 kg P and 60 kg K ha^−1^ was applied at the time of sowing and an additional 60 kg N ha^−1^ fertilizer was applied 4 weeks later. Pre-emergence herbicides (gramazone and atrazine) were sprayed at the rate of 5-L ha^−1^, which was followed by manual weeding to keep the trials weed-free.

### Performance testing under artificial *Striga* infestation and non-infested conditions

All regional hybrid trials evaluated under artificial *S. hermonthica* infestation (STRIN) and non-infested (STRNO) conditions at Kubwa and Mokwa in Nigeria during the main rainy seasons for 8 years were also laid-out in alpha lattice design with two replications. The characteristics of the two testing sites and the complete management of these trials under STRIN and STRNO conditions were described by Menkir et al. (2020). Each hybrid was planted in adjacent infested and non-infested strips facing opposite to each other and separated by 1.5 m alley. Within each strip, the same hybrid was planted in two infested rows and two non-infested rows that were planted directly opposite to each other to determine precise estimates of yield losses due to *S. hermonthica* damage (Kling et al., 2000).

Each hybrid was planted in two 5 m long rows with a spacing of 0.75 m between rows and 0.25 m spacing between plants within a row. The non-infested rows were treated with ethylene gas injected into the soil from a cylinder to stimulate the germination of *Striga* seeds 2 weeks before planting. Every year, *S. hermonthica* seeds used for infestation were collected from sorghum fields around Abuja and Mokwa. Each hole of about 6 cm deep and 10 cm wide where maize seeds were planted was injected with 8.5 g of sand-mixed *S. hermonthica* seed innoculum. The number of germinable *S. hermonthica* seeds placed in each hole was estimated at 3,000. We placed two maize seeds into each hole infested with sand-mixed *S. hermonthica* seeds and covered the holes with soil. One plant was manually removed from each hill 2 weeks after planting (wks) to attain a population density of 53,333 plants ha^−1^. As *S. hermonthica* infection is high under low nitrogen (Kling et al., 2000), nitrogen, phosphorus and potassium were applied at the rate of 30, 60, and 60 kg ha^−1^ at planting, respectively. Additional 30 kg ha^−1^ nitrogen was applied 4 weeks later. Weeds other than *S. hermonthica* were removed by hand throughout the cropping season.

### Performance testing in multi-environment trials under rainfed conditions

The regional elite hybrid trials were also arranged in alpha lattice designs with three replications and were planted during the main rainy seasons in collaboration with the national agricultural research systems (NARS) and private seed companies in 174 test environments representing the diverse maize growing conditions in West Africa (Supplementary Figure S1). Each hybrid was planted in two 5 m long rows each with spacing of 0.75 m between rows and 0.5 m between plants within a row. The collaborators in the NARS and private seed companies used crop management practices, rates of fertilizer application and weed control methods recommended for each of their testing location when they conducted these trials.

### Trait measurements

The traits recorded in each plot under drought stress and well-watered conditions include days to anthesis and silking, anthesis-silking interval, plant and ear heights, number of ears per plant and grain yield. The protocols used to record these traits from regional trials were described by Menkir et al. (2016). Similarly, the detail descriptions of protocols used to record the sets of traits from regional hybrid trials evaluated under *Striga*-infested and non-infested conditions as well as in multiple testing sites by collaborating partners were presented by Menkir et al. (2020). Two additional *Striga*-resistance related traits, namely host plant damage symptom rating and number of emerged parasitic plants, were recorded under *Striga* infestation. Host plant damage symptoms (STDR) were visually rated in each infested row at 8 and 10 weeks after planting (wks) using a scale of 1–9, where 1 = no visible host plant damage symptom and 9 = all leaves completely scorched, resulting in premature death (Kim et al., 2002). The total numbers of emerged *S. hermonthica* plants (STRCO) were also counted in each infested row at 8 and 10 weeks.

In the present study, grain yield recorded under MDS, WW, STRIN, STRNO and in MET, was considered as the primary trait for estimating genetic gains. All ears harvested from each plot were shelled to determine percent moisture, which was used to determine grain yield adjusted to 15% moisture under each growing condition. Grain yield was calculated from shelled grain under MDS and WW, and from ear weight and grain moisture under STRIN, STRNO and in MET assuming a shelling percentage of 80% and final adjusted moisture content of 15% in each testing environment.

### Statistical analyses

We estimated the rate of genetic gain using regional hybrid trials that were routinely conducted at IITA experiment stations and across many locations in collaboration with partners as part of the IITA maize breeding strategy to channel elite products to the national agricultural research system and the private sector. The analysis was conducted for grain yield recorded under STRIN and STRNO conditions and across MET using the model provided by Mackay et al. (2011). In each of these growing conditions, the year and location combinations were treated as environments.

$$y_{ibrjk}=\mu +G_{i}+Y_{k}+R_{rjk}+B_{brjk}+{GY}_{ik}+S_{jk}+e_{irbjk}$$ (1)

where y_irbjk_ is the yield of the *i*th hybrid of the *b*th block nested into the *r*th replication in the *j*th location and *k*th year; μ is the overall mean; G_i_ is the effect of the *i*th hybrid; Y_k_ is the effect of the *k*th year; R_rjk_ is the effect of the *r*th replication in the *j*th location and *k*th year; B_brjk_ is the effect of the *b*th block nested into the *r*th replication in the *j*th location and *k*th year; G_ik_ is the ikth hybrid × year interaction effect; S_jk_ is the effect of location j within year k; e_ijk_ is the residual, attributable to the combined effects of within-trial error and hybrid x location within-year interaction.

For the MDS and WW environments that were conducted in only one location, a similar model was considered where the S_jk_ component was removed along with the j (location) indices.

$$y_{ibrk}=\mu +G_{i}+Y_{k}+R_{rk}+B_{brk}+{GY}_{ik}+e_{irbk}$$ (2)

All effects except μ, G_i_ and Y_k_ were assumed to be random (Mackay et al., 2011). We also assumed heterogeneous residual variances and fitted separate residual variance for each location x year combination for MET, STRIN, and STRNO environments and for each year for MDS and WW environments.

Genetic improvement was assessed to identify a non-genetic trend due to agronomic practices and changing climatic conditions and a genetic trend due to breeding efforts. Because of these two trends, a simple mixed model with independent random genotype and year main effects would potentially yield biased results due to anticipated large, potentially non-linear trends over time for both hybrid and year effects (Mackay et al., 2011). Hence, we adopted a two-stage analysis in which hybrids and years were first treated as fixed factors in a combined analysis within each environment. Analyses of individual trials had been performed to estimate heritability for each of the location x year combinations across MET and under STRIN and STRNO conditions or for each year under MDS and WW conditions. For each individual trial, we used a mixed model with the hybrids as random effects using the method of Cullis et al. (2006), which is suitable for analysis of METs with unbalanced data sets when all genotypes were not grown in all location x year combinations or all years. Two trials conducted under *Striga* non-infested conditions, and 40 trials evaluated in METs having heritability estimates of less than 0.2 were discarded. Trends were then determined in a second stage of analysis by a weighted regression of the estimated hybrids adjusted means on year of origin within each environment. The weights were the inverse of the squared standard errors of the adjusted means of the hybrids. The percent change in genetic gain for each environment (MDS, WW, STRIN, STRNO, and MET) due to genetic causes was estimated as a ratio of the regression slope (in the second stage) to the y-intercept of the regression plus the slope multiplied by the year of first testing.

Genotypic correlations between environments (MET, STRIN, STRNO, MDS and WW) were estimated for grain yield following the method of Malosetti et al. (2013):

$$y_{ip}=\mu +G_{i}+E_{p}+e_{ip}$$ (3)

where y_ip_ is the yield-adjusted mean of the *i*th hybrid in the *p*th environment estimated from the combined analysis using either Model (1) or (2), where G_i_ is the effect of the *i*th hybrid, E_p_ is the effect of environment p, and e_ip_ is the residual. We assume that G_i_ and e_ip_ are mutually independent and distributed as Gaussian, with zero means, such that: $G_{i}\;∼\;N\left(0,\;\sigma_{G}^{2}\right)$ and e_ip_ $e_{ip}\;∼\;N\left(0,\;\sigma_{e_{p}}^{2}\right)$ , hence heterogeneous residual variances. The genetic correlation between two environments is estimated as:

$$r_{\left(p,\;p^{\prime }\right)}=\frac{\sigma_{G}^{2}}{\sqrt{\sigma_{G}^{2}+\sigma_{e_{p}}^{2}}\;\sqrt{\sigma_{G}^{2}+\sigma_{e_{p^{\prime }}}^{2}}}$$ (4)

When traits are correlated, breeding value predictions from a multivariate model can be more accurate than univariate models (Isik et al., 2017). Therefore, we used multivariate mixed models to estimate the genotypic correlations between yield and other traits recorded under MDS and STRIN. All analyses were carried out in R (R Core Team, 2021) using ASReml-R (David, 2020) for fitting the mixed models. To identify traits contributing significantly to yield gains under MDS and STRIN conditions, trait BLUPs were used to perform principal component analysis using the correlation matrix in Statistical Analysis System (SAS) software version 9.4 (SAS Institute, 2016). Correlation coefficients between the original trait BLUPs and their corresponding principal component axis scores were calculated to identify traits contributing significantly to each principal component axis. Yield BLUPs of the hybrids were then regressed against the first principal axis (PC1) scores using PROC REG in SAS (SAS Institute, 2016).

## Results

### Features of regional trials data used for analyzing genetic trends

IITA organized cooperative regional trials involving DTSTR, STRCOM, CONCOM and LOCAL check for 8 years with constant addition of new hybrids and removal of the worst performing ones and shared them with partners for extensive testing. Although the data sets recorded from trials conducted across many years and diverse rained field environments permit periodic assessment of the improvements achieved in a breeding program, at times they may have low connectivity to obtain dependable genetic gain estimates. In the present study, we selected data sets having one STRCOM and three DTSTR hybrids as common genotypes over a period of 8 years to maximize connectivity. In addition, 14 DTSTR, one STRCOM and three CONCOM hybrids were advanced and re-evaluated for five to 7 years making the data sets well connected to preceding testing years. Therefore, the data sets recorded under stressful and favourable growing conditions as well as across diverse field environments had good connectivity for running separate analyses to obtain reliable genetic gain estimates.

### Improvements in grain yield under managed drought stress and full irrigation

**FIGURE 1:**
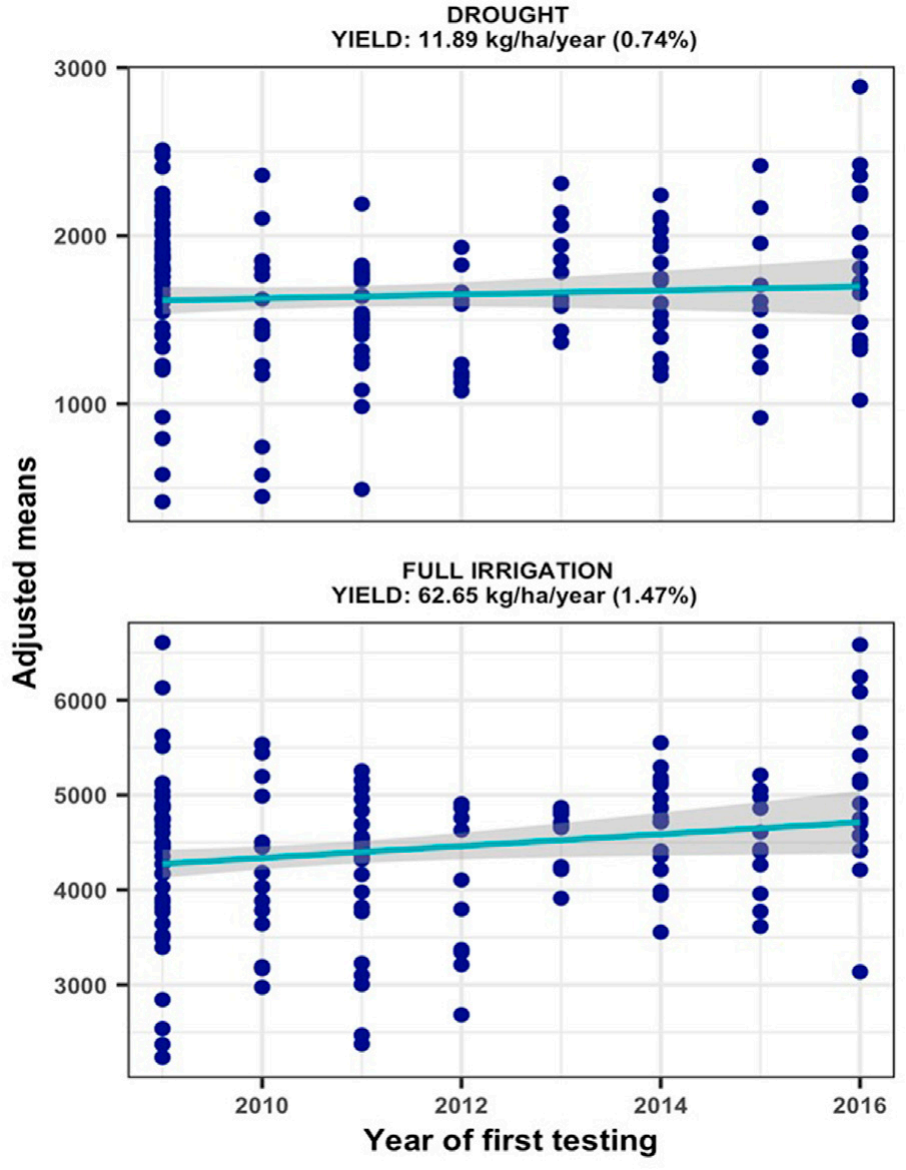
Genetic gain estimates from regional collaborative trials conducted for 8 years under managed drought stress and full irrigation.

The hybrids were exposed to drought stress by withdrawing irrigation from 15 to 20 days before anthesis to physiological maturity. Since appreciable rain was not received from December to end of February at Ikenne (Supplementary Table S3), except for 2013/2014, the hybrids were exposed to water deficit at reproductive and grain filling stages with no additional irrigation applied until harvest. The resulting drought stress induced during these periods resulted in trial mean grain yields varying from 939 to 2988 kg/ha under MDS and from 3588 to 5292 kg/ha under WW conditions. Relative to WW condition, the intensity of drought stress in our study reduced average hybrid yields by 64% in 2012, 17% in 2013, 62% in 2014, 72% in 2015, 80% in 2016, 68% in 2017, 74% in 2018 and 54% in 2019. The yield reductions in seven of the 8 years in our study were comparable to the 40–60% average yield reductions considered appropriate to characterize maize genotypes for drought tolerance (Bänziger et al., 2000). Repeatability estimates for grain yield recorded in individual trials varied from 0.53 to 0.76 under MDS and from 0.63 to 0.90 under WW conditions, which allowed inclusion of all the trials conducted for 8 years in subsequent analyses of genetic trends. The results of regression analyses found significant annual yield increases of 11.89 kg ha^−1^ under MDS (*p = 0.0437*) and 62.65 kg ha^−1^ under WW (*p = 0.0334*) conditions, representing annual yield gains of 0.74% and 1.47%, respectively (Figure 1).

Comparative performance of the DTSTR hybrids relative to STRCOM and CONCOM hybrids as well as the local check is an important reference to assess genetic gains because these checks are the best available cultivars included in regional trials tested over a period of 8 years. As shown in Supplementary Figure S2, mean grain yields of the DTSTR hybrids were higher than those of STRCOM and CONCOM hybrids and the LOCAL check under both MDS and WW conditions. The best 44 DTSTR hybrids tested under MDS for at least 2 years displayed average yield increases of 69% over that of the STRCOM hybrids, 50% over that of the 16 CONCOM hybrids tested for at least 2 years and 180% over that of the LOCAL check (Supplementary Table S3). Again, the best 44 DTSTR hybrids had average yield increases of 23% over that of the STRCOM hybrids, 11% over that of the 16 CONCOM hybrids and 111% over the LOCAL check under WW condition.

### Improvements in grain yield under *Striga* infested and non-infested conditions

**FIGURE 2:**
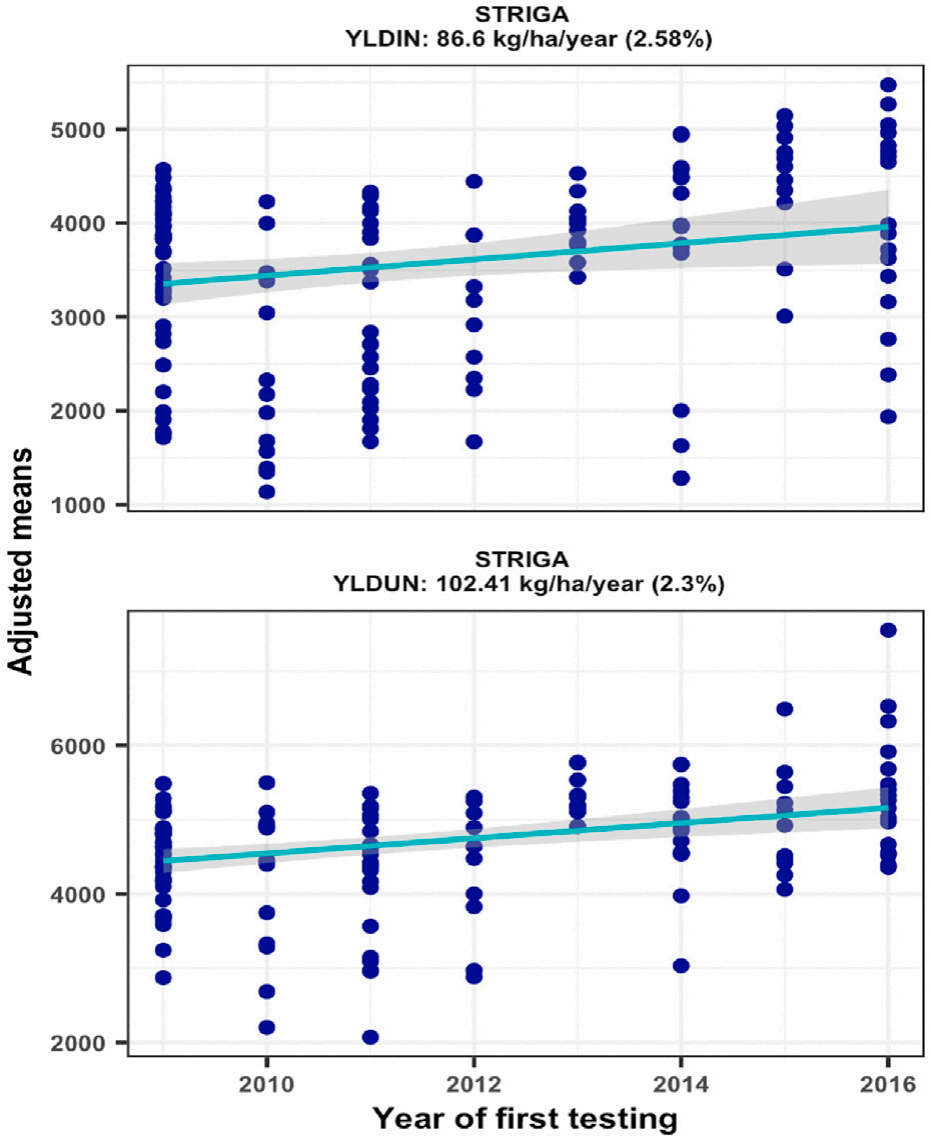
Genetic gain estimates from regional collaborative trials conducted at two locations for 8 years under artificial Striga infestation and non-infested conditions.

**FIGURE 3:**
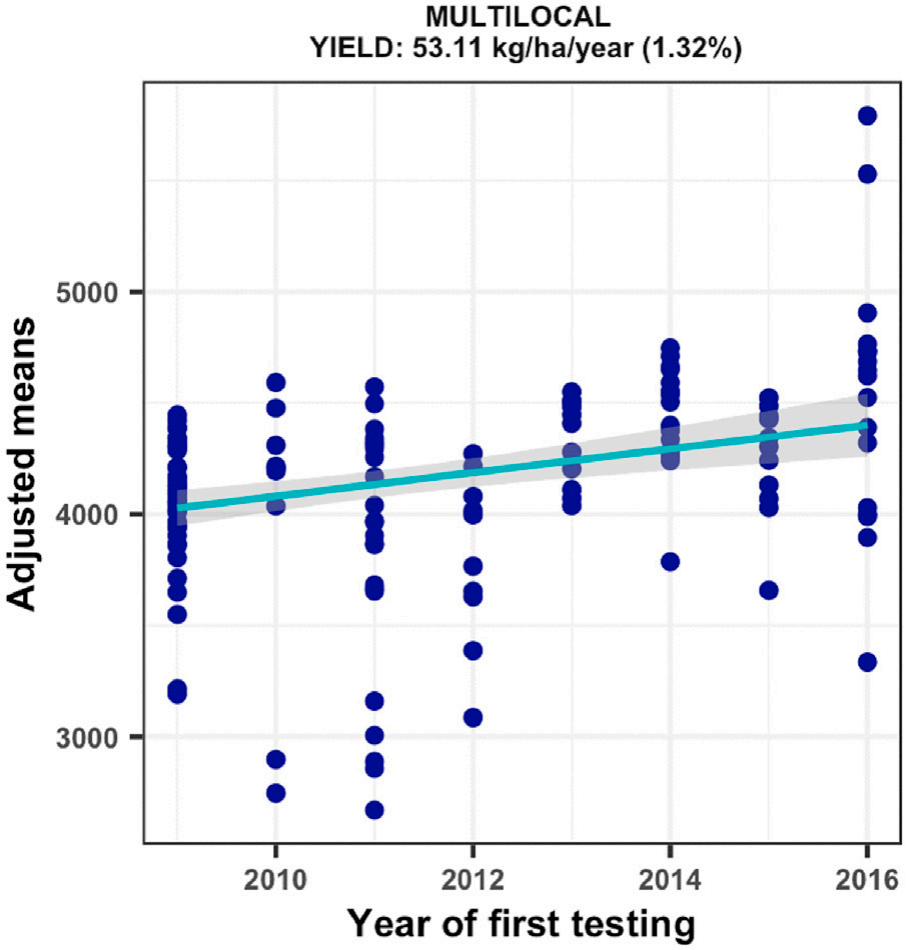
Genetic gain estimates from regional collaborative trials conducted for 8 years across multiple test locations in West Africa.

Trial mean grain yields of the 16 environments varied considerably ranging from 1851 to 6076 kg/ha under *Striga* infestation (STRIN) and from 2573 to 6514 kg/ha under non-infested conditions (STRNO). As most of the hybrids were bred for resistance to *Striga*, the average yield reductions resulting from *Striga* damage ranged from 1% to 39%. Repeatability estimates for the sixteen *Striga* infested environments varied from 0.22 to 0.91 and from 0.02 to 0.81 for non-infested environments. Two environments with repeatability values of less than 0.20 under non-infested conditions were excluded from the genetic trend analysis. As shown in Figure 3, the regression analyses found significant annual yield gains of 2.58% under STRIN (*p = 0.0216*) and 2.30% under STRNO (*p = 0.0001*) condition, representing yield increases of 86.60 and 102.44 kg ha^−1^, respectively (Figure 2).

Yield improvements under STRIN and STRNO condition was assessed by comparing the average grain yield of the DTSTR hybrids relative to those of the STRCOM and CONCOM hybrids and the LOCAL check. As shown in Supplementary Figure S3, the DTSTR hybrids had higher mean grain yields than that of the STRCOM and CONCOM hybrids, and the LOCAL check under both STRIN and STRNO conditions. The best 44 DTSTR hybrids evaluated for at least 2 years outyielded the STRCOM hybrids by 13%, the 16 CONCOM hybrids evaluated for at least 2 years by 118% and the LOCAL check by 133% under STRIN (Supplementary Table S3). Under STRNO condition, the best 44 DTSTR hybrids also displayed yield advantages of 15% relative to the STRCOM hybrids, 19% relative to the 16 CONCOM hybrids and 33% relative to the LOCAL check. The best 44 DTSTR hybrids sustained 38% and 37% less *Striga* damage symptoms in comparison to the 16 CONCOM hybrids and the LOCAL check, respectively (Supplementary Table S3). Relative to the 16 CONCOM hybrids and the LOCAL check, the 44 DTSTR hybrids also supported 36% and 29% less emerged parasites, respectively.

### Improvements in grain yield across rainfed field environments

The regional trials evaluated across a broad range of rainfed field environments displayed mean grain yields varying from 4370–8073 kg ha^−1^. Amongst the 174 test environments, 40 had repeatability values of less than 0.20 for grain yield. The estimated genetic gain using the remaining 134 environments with repeatability estimates of 0.20–0.99 showed a significant ((*p = 0.0001*) annual yield increase of 53.11 kg ha^−1^, representing a relative genetic gain of 1.32% year^−1^ (Figure 3). As shown in Supplementary Figure S4, all DTSTR hybrids had competitive average grain yield to that of all CONCOM hybrids but they outyielded the STRCOM hybrids and the LOCAL check. In addition, the 44 best DTSTR hybrids evaluated for at least 2 years produced as high average grain yield as the 16 CONCOM hybrids but displayed average yield increases of 11% over that of the STRCOM hybrids and 20% over that of the LOCAL check across rainfed field environments (Supplementary Table S3).

### Relationship of grain yield with multiple traits under drought stress and Striga infestation

**FIGURE 4:**
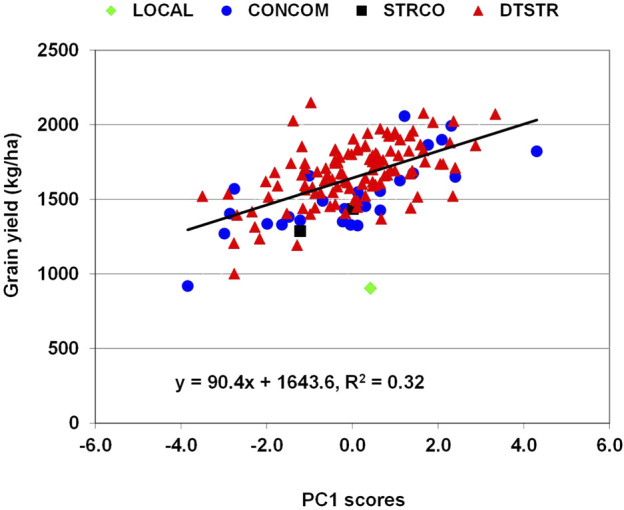
Regression analysis grain yield on PC1 axis scores of hybrids evaluated in regional cooperative trials evaluated under managed drought stress.

**FIGURE 5:**
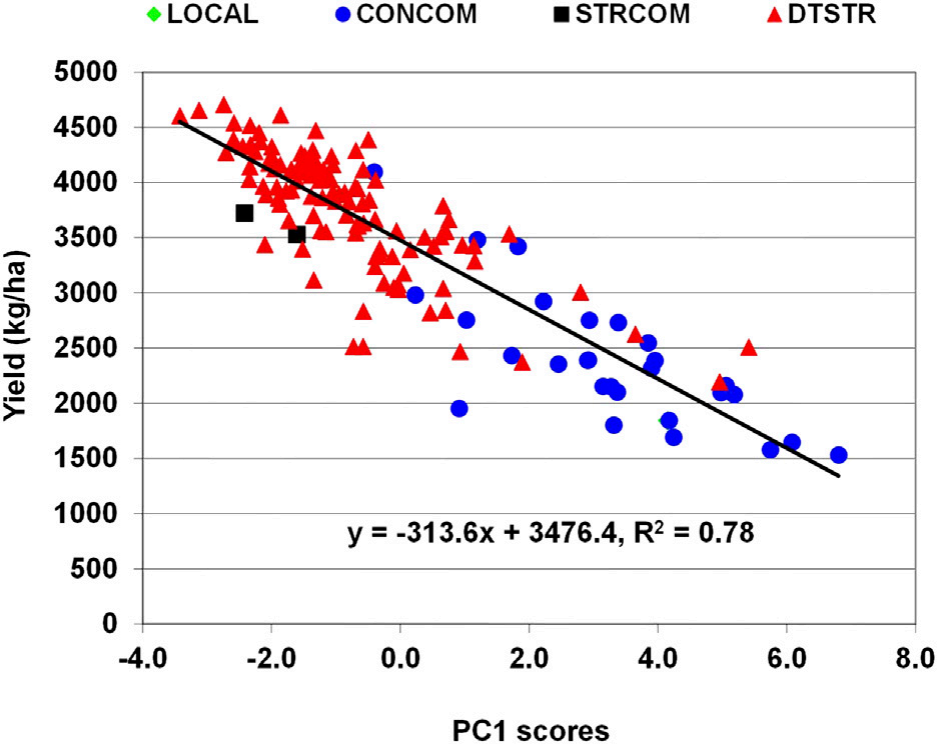
Regression analysis grain yield on PC1 axis scores of hybrids evaluated in regional cooperative trials evaluated under Striga infestation.

In maize, grain yield is a primary trait affected by many stress-related traits. Genetic correlations between grain yield under MDS and anthesis days and anthesis-siling interval were significant and negative, whereas those of yield with plant height, ear height and number of ears per plant were significant and positive (Supplementary Table S4). Significant and negative genetic correlations were also detected between yield under STRIN and anthesis days, silking days, ear aspect scores, *Striga* damage rating and number of emerged *Striga* count at both eight and 10 weeks after planting, while those of yield with plant height and number of ears per plant were significant and positive. Further examination of the relationship of these traits with grain yield using principal component analysis found that the first principal component axes (PC1) alone represented 41% and 55% of the total variations under MDS and STRIN, respectively. Regression analysis showed that an increase in grain yield under MDS was associated with increases in PC1 axis scores, which were correlated with significant reduction in anthesis days and increases in plant height, ear height and number of ears per plant (Supplementary Table S5). As shown in Figure 4, many of the DTSTR hybrids combined positive PC1 scores with higher grain yields under MDS. Under STRIN, the regression analysis found an inverse relationship between grain yield and PC1 axis scores, which were associated with significant delays in anthesis and silking, reduction in plant height and number of ears per plant, and increases in ear aspect scores, *Striga* damage rating and number of emerged parasite count (Supplementary Table S5). Many DTSTR hybrids combined negative PC1 axis scores with higher grain yields (Figure 5).

### Relationships between the different growing conditions

Genetic correlations were computed between grain yields of different growing conditions to examine the effectiveness of the sequential selection scheme in attaining yield improvements under stressful and non-stressful conditions and across diverse field environments. Significant genetic correlations were found between MDS and WW (r_g_ = 0.73 ± 0.04) and between STRIN and STRNO (r_g_ = 0.62 ± 0.05) conditions. The genetic correlations between yields under MDS and STRIN was also significant (r_g_ = 0.41 ± 0.07). Grain yield under MDS and STRNO (r_g_ = 0.60 ± 0.05), STRIN and WW (r_g_ = 0.37 ± 0.07) and STRNO and WW (r_g_ = 0.67 ± 0.05) showed positive and significant genetic correlations. Also, the genetic correlations of yields in MET with MDS (r_g_ = 0.58 ± 0.06), MET and STRIN (r_g_ = 0.44 ± 0.07), MET with WW (r_g_ = 0.71 ± 0.04), and MET with STRNO (r_g_ = 0.76 ± 0.04) were positive and significant.

## Discussions

Maize production fields in tropical savannas experience frequent occurrence of droughts and expansion of *Striga* infestation favoured by increasing temperatures due to climate change, resulting in significant yield losses. IITA in partnership with national agricultural research institutes has thus implemented a sequential selection scheme to develop maize hybrids combining tolerance to drought with resistance to *Striga hermonthica* using reliable screening protocols. The present study used the data recorded in cooperative regional hybrid maize trials conducted for 8 years to estimate genetic gains in grain yield under stressful and non-stressful growing conditions as well as across rainfed field environments. Results of regression analyses found a yield gain of 11.89 kg ha^−1^ year^−1^ under drought stress intensity that reduced average grain yields from 54% to 80% in seven of the 8 years. Although Jiang et al. (2018) reported that the occurrence of drought during the reproductive stage for more than 30 days results in no recovery of maize plants even after irrigation, the yield gain recorded under prolonged water deficit in our study indicates that the parental lines selected for drought tolerance possessed favourable alleles boosting appreciable levels of grain yields in hybrids under both MDS and WW conditions. The yield gain recorded in our study was comparable to that of 14 kg ha^−1^ year^−1^ reported for early maize varieties (Badu-Apraku et al., 2013b), but was lower than the yield gains of 34 kg ha^−1^ year^−1^ for extra-early maize varieties and 46.5 kg ha^−1^ year^−1^ for extra-early maturing hybrids, respectively (Badu-Apraku et al., 2018; Badu-Apraku et al., 2021). The yield gain in our study was also lower than that of 32.5 kg ha^−1^ year^−1^ reported for intermediate-late maturing maize hybrids under MDS in Eastern and Southern Africa (Masuka et al., 2017b). As pointed out by Edmeades et al. (1995), the high rates of genetic gains in the early flowering genotypes could originate from sustenance of less stress at flowering time than the intermediate-late flowering genotypes included in our study.

The low genetic gain recorded under MDS in our study could also arise form a complex interplay of year-to-year variation in rainfall, temperature, and humidity during trial evaluation interacting with variation in flowering time and water holding capacity of the soil, impacting on growth and performance of hybrids across environments (Bänziger and Cooper, 2001). Crespo-Herrera et al. (2017) found significant genotype × environment interaction in wheat that led to lower genetic gain estimates. Less genetic gain in grain yield could also be recorded under MDS due to the broad range of physiological responses of the maize hybrids to drought stress triggered by the timing, duration, and severity of specific water deficits (Snowdon et al., 2021). Moreover, the use of *Striga* resistant populations improved through S1 recurrent selection without infusion of tropical donor lines having superior drought tolerance genes as sources of parents the DTSTR hybrids could contribute to the low yield gains recorded under MDS.

Given the increasing demand for more maize grain and the anticipated adverse effects of climate variability and change, the genetic gain achieved under MDS in our study is not sufficient to achieve the production level that is necessary to meet the projected demand for maize grain in sub-Saharan Africa (Santpoort, 2020; Jayne and Sanchez, 2021). To realize higher rates of genetic gain in grain yield under both MDS and STRIN, breeders need to adjust the current selection scheme by crossing tropical elite drought tolerant donor lines with existing elite DTSTR lines to form source populations for new inbred line development. The resulting source populations can be used to develop numerous early generation lines that can be screened first under MDS coinciding with flowering period and advanced at high selection intensity followed by screening the selected lines under artificial *Striga* infestation to generate new maize inbred lines with tolerance to stress combinations. Further accumulation of such beneficial drought tolerant allelic combinations may drive the next jump in genetic gains for grain yield in the future.

The elite DTSTR hybrids involving parental lines derived from *Striga* resistant source populations were repeatedly evaluated under both *Striga* infested and non-infested conditions before they were included in regional trials. Since the artificial infestation protocol used during inbred line development and hybrid evaluation across 8 years did not change, it provided consistently high levels of parasite infection that had significant effects on attaining higher rate of genetic gain in grain yield particularly under STRIN. The genetic gain of 86.60 kg ha^−1^ year^−1^ recorded under STRIN in our study was higher than that of 41 kg ha^−1^ year^−1^ for extra-early and 42 kg ha^−1^ year^−1^ for early maturing open-pollinated maize varieties (Badu-Apraku et al., 2013a, 2016), but was lower than that of 93.7 kg ha^−1^ year^−1^ for intermediate-late and 101 kg ha^−1^ year^−1^ for early maturing hybrids recorded under STRIN (Menkir and Meseka, 2019; Badu-Apraku et al., 2021). The genetic gain of 102.44 kg ha^−1^ year^−1^ recorded under STRNO in our study was higher than that of 34 kg ha^−1^ year^−1^ for early and 54 kg ha^−1^ year^−1^ for extra-early maturing open-pollinated maize varieties, 29.3 kg ha^−1^ year^−1^ for intermediat-late and 61.0 kg ha^−1^ year^−1^ for early matuing hybrids obtained under STRNO conditions (Menkir and Meseka, 2019; Badu-Apraku et al., 2021). It thus appears that the *Striga* screening protocol was effective in selecting and accumulating favourable *Striga* resistance alleles that enhanced the capacity of maize plants to sustain less parasite damage and suppress *Striga* emergence to attain high productivity gains under STRIN. The resulting improvements in the overall plant growth with selection under STRIN could also increase the rate of photosynthesis leading to increased rates of yield gain observed even under STRNO conditions.

Understanding the impact of controlled stress screening protocols on realized yield gains across variable rainfed conditions is important to develop hybrids that can be successfully commercialized in the target production environments. In our study, a modest yield gain of 53.11 kg ha^−1^ year^−1^ was realized notwithstanding the absence of direct selection for broad adaptation across diverse rainfed field growing conditions. These results suggest that simultaneous selection for tolerance to drought and resistance to S*triga*, while also assessing performance under non-stressful conditions, may lead to constitutive changes that are also beneficial in achieving increased yield gains in target environments where varying climatic conditions, soil properties and management practices commonly occur in smallholder farmers’ fields. Bänziger et al. (2002) proposed that use of reliable screening protocols could increase both specific and broad adaptations to unpredictable occurrence of stresses leading to the delivery of yield gains to smallholder farmers.

The success in achieving improvements for tolerance to stress combinations depends on the capacity to select hybrids that are superior in performance under stressful conditions and competitive in grain yields with commercial hybrids as well across diverse rainfed environments. May of the hybrids evaluated in the present study that produced higher grain yields under both manged drought stress and artificial *Striga* infestation were also competitive or had better grain yields than the *Striga* resistant and conventional commercial hybrids across diverse field environments. The sequential breeding scheme has thus demonstrated a significant advantage in continuously delivering better drought tolerant and *Striga* resistant hybrids to the national partners in Africa without compromising yields across varying rainfed growing conditions. The national partners and seed companies have already identified promising hybrids from the regional trials for further testing and eventual commercialization. Such hybrids would provide dependable yields to rural communities and reduce chronic food deficits in production areas adversely affected by simultaneous presence of drought and *S. hermonthica*.

Genetic gain in grain yield under MDS condition was correlated with early flowering, better synchrony between male and female flowering time, taller plants, and increased number of ears, suggesting that the observed yield gain was primarily determined by changes in plant growth and reproductive structures. These could lead to increased capacity of maize plants to remobilize carbohydrates stored in the steam and enhance transfer of assimilates to the developing ears (Edmeades et al., 1995; Baenziger et al., 1999). Our findings are consistent with others who concluded that a shorter anthesis silking interval, increased biomass and reduced barrenness under drought stress are associated with high yield gain in maize (Bolaños and Edmeades, 1993; Byrne et al., 1995; Baenziger et al., 1999). The realization of higher yield gain under STRIN was associated with less parasite damage to the leaves, stems, and ears, which improved the supply of photosynthate to the developing ears under *Striga* infestation. Moreover, genetic gain under STRIN was related to less emerged parasites, which reduced water, nutrient and photosynthate acquisition by the parasite form maize plants that could result in higher grain yields. It is thus more likely that the selection scheme had a positive impact on improving the major agronomic traits that increased the overall genetic gain under stressful conditions.

We found significant and positive genetic correlations between grain yields measured under MDS and STRIN and those recorded under each specific stress with yields under non-stress conditions. In addition, the genetic correlations of grain yield recorded under each stress or non-stress condition with grain yield obtained in MET were significant and positive. It is thus likely that the response to selection for drought tolerance and resistance to *S. hermonthica* shared common adaptation mechanisms at the molecular, biochemical, and physiological levels conferring tolerance to both specific and multiple stresses that led to yield gains for specific stress type and across a broad range of production environments as well (Atkinson and Urwin, 2012; Malenica et al., 2021). Studies found stress-responsive genes expressed under combined abiotic and biotic stresses possibly inducing common physiological and biochemical mechanisms to activate defence responses of crop plants to multiple biotic and abiotic stresses (Kissoudis et al., 2014; Pandey et al., 2015; Ramegowda and Senthil-Kumar, 2015; Sun et al., 2015).

In summary, screening lines under carefully managed stress conditions provided adequate intensity of drought stress and parasite infection for achieving yield gains under stressful and favourable growing conditions. These yield gains were accompanied by improvements in grain yield across naturally occurring stressful and favourable growing environments in the savannas during the main cropping season. Consequently, direct selection for grain yield and key other traits under manged stress and non-stress conditions was effective in increasing both specific and broad adaptation of the DTSTR hybrids to unpredictable occurrence of stresses prevailing in smallholder farmers’ fields. The next quantum jump in yield gains under both managed drought stress and *Striga* infestation may be achieved by infusing novel sources of drought tolerant genes into the existing elite drought tolerant and *Striga* resistant lines. Ccharacterizing the underlying genetic and physiological mechanisms using the drought tolerant and *Striga* resistant lines may also facilitate the development of novel tools to accelerate the rate of genetic gains under stress combinations that are frequently encountered by smallholder farmers.

## Data Availability

The original contributions presented in the study are included in the article/Supplementary Material, further inquiries can be directed to the corresponding author.
