# Supplementary material for: Estimating genetic gains for tolerance to stress combinations in tropical maize hybrids
Source: Front Genet. 2022 Dec 8;13:1023318. doi: 10.3389/fgene.2022.1023318 (PMC9779929; doi:10.3389/fgene.2022.1023318)
Supplement: Supplementary file 1 [file Table1.DOC]

Supplementary Table S1. List of hybrids included in the regional trials evaluated under stressful and favourable growing conditions as well as in divers rainfed field environments for five years

| Hybrids | Pedigree |
| --- | --- |
| H01 | ACRSYN-W-S2-173-B*4/TZLCompIC4S1-37-5-BBB/IWD-SYN-STR-C3--47-1-BB |
| H02 | ZDiploBC4-19-4-1-#-3-1-B-1-B*4/TZLCompIC4S1-37-1-B*4/IWD-SYN-STR-C3--52-1-BB |
| H03 | ZDiploBC4-19-4-1-#-3-1-B-1-B*4/TZLCompIC4S1-37-1-B*4/IWD-SYN-STR-C3--53-2-BB |
| H04 | ACRSYN-W-S2-173-B*4/TZLCompIC4S1-37-1-B*4/IWD-SYN-STR-C3--53-2-BB |
| H05 | ACRSYN-W-S2-173-B*4/TZLCompIC4S1-37-1-B*4/IWD-SYN-STR-C3--55-3-BB |
| H06 | ACRSYN-W-S2-173-B*4/TZLCompIC4S1-37-1-B*4/IWD-SYN-STR-C3-53-2-B*5 |
| H07 | ZDiploBC4-472-2-2-1-2-3-B-1-B*5/TZLCompIC4S1-37-5-BBB/IWD-SYN-STR-C3--53-2-BB |
| H08 | ZdiploBC4-472-2-2-1-2-3-B*6/TZLCompIC4S1-37-5-BBB/IWD-SYN-STR-C3--55-3-BB |
| H09 | ZDiploBC4-19-4-1-#-3-1-B-1-B*4/TZLCompIC4S1-37-5-BBB/IWD-SYN-STR-C3--55-3-BB |
| H10 | ZDiploBC4-472-2-2-1-2-3-B-1-B*5/TZLCompIC4S1-37-5-BBB/IWD-SYN-STR-C3--67-1-BB |
| H11 | ACRSYN-W-S2-173-B*4/TZLCompIC4S1-37-5-BBB/IWD-SYN-STR-C3--32-2-BB |
| H12 | ZDiploBC4-19-4-1-#-3-1-B-1-B*4/TZLCompIC4S1-37-5-BBB/IWD-SYN-STR-C3--50-2-BB |
| H13 | ACRSYN-W-S2-173-B*4/TZLCompIC4S1-37-1-B*4/IWD-SYN-STR-C3--52-1-BB |
| H14 | ZdiploBC4-472-2-2-1-2-3-B*6/TZLCompIC4S1-37-5-BBB/IWD-SYN-STR-C3--52-4-BB |
| H15 | ZdiploBC4-472-2-2-1-2-3-B*6/(ACRSYN-W-S2-173-B*4/TZLCompIC4S1-37-5-BBB)-4-B/IWD-SYN-STR-C3--70-2-B-B |
| H16 | ZDiploBC4-472-2-2-1-2-3-B-1-B*5/TZLCompIC4S1-37-5-BBB/IWD-SYN-STR-C3--55-3-BB |
| H17 | ((ZDiploBC4-472-2-2-1-2-3-B-1-B*5/ZDiploBC4-19-4-1-#-3-1-B-1-B*4)-25-1-BB/(ACRSYN-W-S2-173-B*4/TZLCompIC4S1-37-1-B*4)-36-B*4)/IITATZISTR1133 |
| H18 | ((ZDiploBC4-472-2-2-1-2-3-B-1-B*5/ZDiploBC4-19-4-1-#-3-1-B-1-B*4)-43-1-BB/(ACRSYN-W-S2-173-B*4/TZLCompIC4S1-37-5-BBB)-3-B*4)/IITATZISTR1133 |
| H19 | (ZDiploBC4-472-2-2-1-2-3-B-1-B*5/ZDiploBC4-19-4-1-#-3-1-B-1-B*4)-25-1-BB/(ACRSYN-W-S2-173-B*4/TZLCompIC4S1-37-5-BBB)-38-1-1-BB/IWD-SYN-STR-C3-52-1-B*5 |
| H20 | (ZDiploBC4-472-2-2-1-2-3-B-1-B*5/ZDiploBC4-19-4-1-#-3-1-B-1-B*4)-2-1-BB/(ACRSYN-W-S2-173-B*4/TZLCompIC4S1-37-5-BBB)-4-B*4/IWD-SYN-STR-C3-52-1-B*5 |
| H21 | ZdiploBC4-472-2-2-1-2-3-B*6/(ACRSYN-W-S2-173-B*4/TZLCompIC4S1-37-1-B*4)-57-B/IWD-SYN-STR-C3--70-2-B |
| H22 | ZdiploBC4-472-2-2-1-2-3-B*6/(ACRSYN-W-S2-173-B*4/TZLCompIC4S1-37-5-BBB)-4-B/IWD-SYN-STR-C3--70-2-B |
| H23 | ZeaDiploBC4-WC3-29-3-1-B*4/(ACRSYN-W-S2-173-B*4/TZLCompIC4S1-37-5-BBB)-27-B/IWD-SYN-STR-C3--32-2-BB |
| H24 | (ZDiploBC4-472-2-2-1-2-3-B-1-B*5/ZDiploBC4-19-4-1-#-3-1-B-1-B*4)-25-1-BB/(ACRSYN-W-S2-173-B*4/TZLCompIC4S1-37-5-BBB)-56-B*4/IWD-SYN-STR-C3-52-1-B*5 |
| H25 | ZDiploBC4-19-4-1-#-3-1-B-1-B*4/(ACRSYN-W-S2-173-B*4/TZLCompIC4S1-37-1-B*4)-16-B/(IITATZI1872 |
| H26 | (1393/Z.Diplo.BC4-19-4-1-#-3-1-B-1-B*4)-46-B-B-B-B/(ACRSYN-W-S2-173-B-B-B-B/TZL Comp. IC4 S1-37-5-B-B-B)-31-1-1-B-B/IITATZISTR1129 |
| H27 | ACRSYN-W-S2-173-B*6/TZLCompIC4S1-37-5-BBB/IWD-SYN-STR-C3-32-1-B*5 |
| H28 | ZdiploBC4-472-2-2-1-2-3-B*8/TZLCompIC4S1-37-5-B*5/IWD-SYN-STR-C3--32-1-B*5 |
| H29 | ACRSYN-W-S2-173-B*6/TZLCompIC4S1-37-5-BBB/IWD-SYN-STR-C3-46-5-B*5 |
| H30 | ZdiploBC4-472-2-2-1-2-3-B*6/(ACRSYN-W-S2-173-B*4/TZLCompIC4S1-37-5-BBB)-4-B/(IITATZI1872 |
| H31 | ZeADiploBC4-WC3-29-3-1-B*4/(ACRSYN-W-S2-173-B*4/TZLCompIC4S1-37-5-BBB)-27-B/IITATZISTR1129 |
| H32 | ZdiploBC4-376-1-1-#-3-1-B-2-BBB/ACR97TZL-CCOMP1-Y-S3-34-3-BBB/ACR97SYN-Y-S1-76-B-B-B-B |
| H33 | ACRSYN-W-S2-173-B*4/TZLCompIC4S1-37-1-B*4/IWD-SYN-STR-C3--32-2-BB |
| H34 | ZDiploBC4-19-4-1-#-3-1-B-1-B*4/TZLCompIC4S1-37-1-B*4/IWD-SYN-STR-C3--67-1-BB |
| H35 | ACRSYN-W-S2-173-B*4/TZLCompIC4S1-37-5-BBB/IWD-SYN-STR-C3-55-3-B*5 |
| H36 | ACRSYN-W-S2-173-B*4/TZLCompIC4S1-37-5-BBB/IWD-SYN-STR-C3--67-1-BB |
| H37 | ZDiploBC4-472-2-2-1-2-3-B-1-B*5/TZLCompIC4S1-37-5-BBB/IWD-SYN-STR-C3--32-2-BB |
| H38 | ACRSYN-W-S2-173-B*4/TZLCompIC4S1-37-5-BBB/IWD-SYN-STR-C3--52-4-BB |
| H39 | ACRSYN-W-S2-173-B*4/TZLCompIC4S1-37-5-BBB/IWD-SYN-STR-C3--53-2-BB |
| H40 | ZDiploBC4-472-2-2-1-2-3-B-1-B*5/TZLCompIC4S1-37-1-B*4/IWD-SYN-STR-C3--47-1-BB |
| H41 | ZDiploBC4-472-2-2-1-2-3-B-1-B*5/TZLCompIC4S1-37-1-B*4/IWD-SYN-STR-C3--50-2-BB |
| H42 | ACRSYN-W-S2-173-B*4/TZLCompIC4S1-37-1-B*4/IWD-SYN-STR-C3--52-4-BB |
| H43 | ZDiploBC4-19-4-1-#-3-1-B-1-B*4/ACRSYN-W-S2-173-B*4/IWD-SYN-STR-C3--52-1-B*5 |
| H44 | ZdiploBC4-472-2-3-4-3-B-2-B*5/TZLCompIC4S1-37-5-BBB/IWD-SYN-STR-C3--70-1-B*5 |
| H45 | ACRSYN-W-S2-173-B*4/TZLCompIC4S1-37-5-BBB/IWD-SYN-STR-C3--52-4-BB |
| H46 | ZdiploBC4-472-2-2-1-2-3-B*6/TZLCompIC4S1-37-1-B*4/IWD-SYN-STR-C3--70-1-BB |
| H47 | ZDiploBC4-472-2-2-1-2-3-B-1-B*5/TZLCompIC4S1-37-1-B*4/IWD-SYN-STR-C3--70-1-BB |
| H48 | ZDiploBC4-19-4-1-#-3-1-B-1-B*4/TZLCompIC4S1-37-5-BBB/IWD-SYN-STR-C3--32-2-BB |
| H49 | ((1393/Z.Diplo.BC4-19-4-1-#-3-1-B-1-B*4)-61-1-1-B-B/(ACRSYN-W-S2-173-B-B-B-B/TZL Comp. IC4 S1-37-1-B-B-B-B)-54-B-B-B-B))/IITATZISTR1117 |
| H50 | ((Z. Diplo.BC4-472-2-2-1-2-3-B-1-B-B-B-B-B/Z.Diplo.BC4-19-4-1-#-3-1-B-1-B-B-B-B)-2-1-B-B/(ACRSYN-W-S2-173-B-B-B-B/TZL Comp. IC4 S1-37-5-B-B-B)-4-B-B-B-B)/IITATZISTR1134 |
| H51 | ((Z. Diplo.BC4-472-2-2-1-2-3-B-1-B-B-B-B-B/Z.Diplo.BC4-19-4-1-#-3-1-B-1-B-B-B-B)-25-1-B-B/(ACRSYN-W-S2-173-B-B-B-B/TZL Comp. IC4 S1-37-5-B-B-B)-38-1-1-B-B)/IITATZISTR1134 |
| H52 | (1393/ZDiploBC4-19-4-1-#-3-1-B-1-B*4)-61-1-1-BB/(ACRSYN-W-S2-173-B*4/TZLCompIC4S1-37-1-B*4)-54-B*4/IITATZISTR1117 |
| H53 | ZDiploBC4-472-2-2-1-2-3-B-1-B*5/(ACRSYN-W-S2-173-B*4/TZLCompIC4S1-37-5-BBB)-35-B/IWD-SYN-STR-C3--32-2-BB |
| H54 | (1393/ZDiploBC4-19-4-1-#-3-1-B-1-B*4)-12-B*4/(ACRSYN-W-S2-173-B*4/TZLCompIC4S1-37-5-BBB)-17-B*4/IWD-SYN-STR-C3--1-1-B*5 |
| H55 | (ZDiploBC4-472-2-2-1-2-3-B-1-B*5/ZDiploBC4-19-4-1-#-3-1-B-1-B*4)-26-1-BB/(ACRSYN-W-S2-173-B*4/TZLCompIC4S1-37-1-B*4)-50-B*4/IWD-SYN-STR-C3--47-1-B*5 |
| H56 | (ZDiploBC4-472-2-2-1-2-3-B-1-B*5/ZDiploBC4-19-4-1-#-3-1-B-1-B*4)-2-1-BB/(ACRSYN-W-S2-173-B*4/TZLCompIC4S1-37-5-BBB)-4-B*4/IITATZISTR1134 |
| H57 | (ZDiploBC4-472-2-2-1-2-3-B-1-B*5/ZDiploBC4-19-4-1-#-3-1-B-1-B*4)-26-1-BB/(ACRSYN-W-S2-173-B*4/TZLCompIC4S1-37-5-BBB)-40-B*4/IWD-SYN-STR-C3--52-1-B*5 |
| H58 | ZDiploBC4-19-4-1-#-3-1-B-1-B*6/TZLCompIC4S1-37-5-B*5/IWD-SYN-STR-C3--32-2-BB |
| H59 | ZdiploBC4-472-2-2-1-2-3-B*8/TZLCompIC4S1-37-5-B*5/IWD-SYN-STR-C3--32-2-BB |
| H60 | (ZDiploBC4-472-2-2-1-2-3-B-1-B*5/ZDiploBC4-19-4-1-#-3-1-B-1-B*4)-2-1-BB/(ACRSYN-W-S2-173-B*4/TZLCompIC4S1-37-5-BBB)-4-B*4/IITATZISTR1134 |
| H61 | 4001xB73LPAx4001-6-2-3-BBB/ACR97TZL-CCOMP1-Y-S3-24-1-B*4 |
| H62 | ZdiploBC4-376-1-1-#-3-1-B-2-BBB/ACR97TZL-CCOMP1-Y-S3-34-3-BBB/ACR97SYN-Y-S1-76-B*4 |
| H63 | (1393/ZDiploBC4-19-4-1-#-3-1-B-1-B*4)-40-BB/IWD-SYN-STR-C3--50-2-BBB/TZLCompIC4S1-37-1-B*6 |
| H64 | ZDiploBC4-19-4-1-#-3-1-B-1-B*4/(ACRSYN-W-S2-173-B*4/TZLCompIC4S1-37-1-B*4)-16-B/IWD-SYN-STR-C3--70-2-B |
| H65 | ZDiploBC4-19-4-1-#-3-1-B-1-B*4/(ACRSYN-W-S2-173-B*4/TZLCompIC4S1-37-1-B*4)-32-B/IWD-SYN-STR-C3--70-2-B |
| H66 | STRLowEmergPoolCOS3670-1-5-3-3-B-1-B*7/(ACRSYN-W-S2-173-B*4/TZLCompIC4S1-37-5-BBB)-3-B/IWD-SYN-STR-C3--70-2-B |
| H67 | Acr.Syn-W S2-173-B-B-B/Z.Diplo BC4-472-2-3-4-3-B-2-B/TZLCompIC4S1-37-1-B*6 |
| H68 | TZLCompIC4S1-38-5-B*6/ACRSYN-W-S2-173-B*7/IWD-SYN-STR-C3--50-2-B-B |
| H69 | 0601-6STR/IWD-SYN-STR-C3--52-4-B*5 |
| H70 | (ZDiploBC4-472-2-2-1-2-3-B-1-B*5/ZDiploBC4-19-4-1-#-3-1-B-1-B*4)-44-1-BB-B/(TZECOMP5-Y-C7-S3-150-B*4/TZECOMP5-25-1-1-3-#-2-B*4)-40-1-BB-B/IWD-SYN-STR-C3-67-1-B*5 |
| H71 | Acr.Syn-W S2-173-B-B-B/Z.Diplo BC4-472-2-3-4-3-B-2-B/TZLCompIC4S1-37-5-B*6 |
| H72 | Z.diplo.BC4-472-2-2-1-2-3-B-B-B-B-B-B/(ACRSYN-W-S2-173-B-B-B-B/TZL Comp. IC4 S1-37-5-B-B-B)-4-B/IWD-SYN-STR-C3--70-2-B |
| H73 | (Z. Diplo.BC4-472-2-2-1-2-3-B-1-B-B-B-B-B/Z.Diplo.BC4-19-4-1-#-3-1-B-1-B-B-B-B)-2-1-B-B/(ACRSYN-W-S2-173-B-B-B-B/TZL Comp. IC4 S1-37-5-B-B-B)-4-B-B-B-B/IWD-SYN-STR-C3--52-1-B-B |
| H74 | (Z. Diplo.BC4-472-2-2-1-2-3-B-1-B-B-B-B-B/Z.Diplo.BC4-19-4-1-#-3-1-B-1-B-B-B-B)-25-1-B-B/(ACRSYN-W-S2-173-B-B-B-B/TZL Comp. IC4 S1-37-5-B-B-B)-56-B-B-B-B/IWD-SYN-STR-C3--52-1-B-B |
| H75 | ZDiploBC4-472-2-2-1-2-3-B-1-B*7/TZLCompIC4S1-37-1-B*6/IWD-SYN-STR-C3-67-1-B*5 |
| H76 | ZdiploBC4-472-2-2-1-2-3-B*8/TZLCompIC4S1-37-5-B*5/IWD-SYN-STR-C3-67-1-B*5 |
| H77 | ACRSYN-W-S2-173-B-B-B-B-B-B/TZLCompIC4S1-37-5-B*5/IWD-SYN-STR-C3-67-1-B*5 |
| H78 | (ZDiploBC4-472-2-2-1-2-3-B-1-B*5/ZDiploBC4-19-4-1-#-3-1-B-1-B*4)-44-1-BB-B/(ACRSYN-W-S2-173-B*4/TZLCompIC4S1-37-5-BBB)-4-B*4-B/IWD-SYN-STR-C3--70-2-B |
| H79 | ACRSYN-W-S2-173-B-B-B-B-B-B/TZL Comp. IC4 S1-37-1-B-B-B-B/IWD-SYN-STR-C3-32-2-B*5 |
| H80 | ACRSYN-W-S2-173-B*4/TZLCompIC4S1-37-5-BBB/IWD-SYN-STR-C3-52-3-B |
| H81 | ZDiploBC4-19-4-1-#-3-1-B-1-B*4/TZLCompIC4S1-37-5-BBB/IWD-SYN-STR-C3-53-2-B-B |
| H82 | ZDiploBC4-472-2-2-1-2-3-B-1-B*5/TZLCompIC4S1-37-1-B*4/IWD-SYN-STR-C3--70-2-B |
| H83 | ACRSYN-W-S2-173-B*4/TZLCompIC4S1-37-5-BBB/IWD-SYN-STR-C3-52-4-B*5 |
| H84 | ZDiploBC4-19-4-1-#-3-1-B-1-B*7/TZLCompIC4S1-37-5-B*6/IWD-SYN-STR-C3--70-2-B-B |
| H85 | TZISTR1199/TZISTR1161/IWD-SYN-STR-C3-18-1-B-B-B |
| H86 | TZISTR1192/TZISTR1128/IWD-SYN-STR-C3-53-2-B*5 |
| H87 | TZISTR1259/TZISTR1223/ACR97TZL-CCOMP1-Y-S3-12-2-B*8 |
| H88 | TZISTR1273/TZISTR1246/ACR97TZL-CCOMP1-Y-S3-12-2-B*8 |
| H89 | TZISTR1273/TZISTR1248/ACR97TZL-CCOMP1-Y-S3-12-2-B*8 |
| H90 | ACRSYN-W-S2-173-B*4/TZLCompIC4S1-37-5-BBB/IWD-SYN-STR-C3--55-3-BB |
| H91 | Syn-Y-STR-(43-2)-1-1-5-1-B*6/Z.diplo.BC4-376-1-1-#-3-1-B-2-B-B/(ACR97SYN-Y-S1-79-B*4/ACR97TZLComp1-YS155-4-1-3-B*4)-66-1-BB-B |
| H92 | Syn-Y-STR-(43-2)-1-1-5-1-B*6/Z.diplo.BC4-376-1-1-#-3-1-B-2-B-B/(ACR97SYN-Y-S1-79-B*4/ACR97TZLComp1-YS155-4-1-3-B*4)-9-1-BB-B |
| H93 | TZISTR1268/TZISTR1223/TZISTR1029 |
| H94 | TZISTR1190/TZISTR1157/TZISTR1136 |
| H95 | TZISTR1199/TZISTR1175/TZISTR1871 |
| H96 | ZDiploBC4-19-4-1-#-3-1-B-1-B*4/TZLCompIC4S1-37-5-BBB/TZISTR1137 |
| H97 | 1001-9STR/IWD-SYN-STR-C3-53-2-B*5 |
| H98 | ZdiploBC4-472-2-2-1-2-3-B*6/TZLCompIC4S1-37-1-B*4/TZISTR1134 |
| H99 | 0804-7STR/IWD-SYN-STR-C3-52-4-B*5 |
| H100 | TZISTR1190/TZISTR1154/TZISTR1134 |
| H101 | IITATZISTR1135/ACRSYN-W-S2-173-B*4/TZLCompIC4S1-37-5-BBB |
| H102 | IITATZISTR1137/ZDiploBC4-19-4-1-#-3-1-B-1-B*4/TZLCompIC4S1-37-5-BBB |
| H103 | IWD-SYN-STR-C3--70-2-B/ZdiploBC4-472-2-3-4-3-B-2-B*8/TZL Comp. IC4 S1-37-5-B-B-B |
| H104 | IWD-SYN-STR-C3--52-4-B*5/TZISTR1179/TZISTR1107 |
| H105 | IWD-SYN-STR-C3--52-2-B/(ACRSYN-W-S2-173-B*4/TZLCOMP1C4-S1-37-1-B*4)DH43-B/(IWDC3SYN/(WhiteDTSTRSyn/IWDC3SYN)-146-BBB-1 |
| H106 | IWD-SYN-STR-C3--52-2-B/(ACRSYN-W-S2-173-B*4/TZLCOMP1C4-S1-37-1-B*4)DH43-B/(IWDC3SYN/(WhiteDTSTRSyn/IWDC3SYN)-146-BBB-1 |
| H107 | TZISTR1137/(ACRSYN-W-S2-173-B*4/TZLCOMP1C4-S1-37-1-B*4)DH10-B/(IWDC3SYN/(WhiteDTSTRSyn/IWDC3SYN)-23-BBB-3 |
| H108 | (ACRSYN-W-S2-173-B*4/TZLCOMP1C4-S1-37-1-B*4)DH19-B/(IWDC3SYN/(WhiteDTSTRSyn/IWDC3SYN)-23-BBB-3/TZISTR1137 |
| H109 | (ACRSYN-W-S2-173-B*4/TZLCOMP1C4-S1-37-5-B*4)DH13-B/(TZLCOMP1-WC6/(WhiteDTSTRSyn/TZLCOMP1-W))-259-BBB-1/TZISTR1152 |
| H110 | TZISTR1161/TZISTR1136/TZISTR1129 |
| H111 | (ACR97SYN-Y-S1-79-B*4/ACR97TZLComp1-YS155-4-1-3-B*4)-9-1-BB-B/Syn-Y-STR-(43-2)-1-1-5-1-B*6/Z.diplo.BC4-376-1-1-#-3-1-B-2-B-B |
| H112 | IWD-SYN-STR-C3-53-2-B*5/IWD-SYN-STR-C3--14-2-B*6/(ACRSYN-W-S2-173-B*4/TZLCompIC4S1-37-1-B*4)-21-B*5 |
| H113 | STRCOM01 |
| H114 | STRCOM02 |
| H115 | CONCOM01 |
| H116 | CONCOM02 |
| H117 | CONCOM03 |
| H118 | CONCOM04 |
| H119 | CONCOM05 |
| H120 | CONCOM06 |
| H121 | CONCOM07 |
| H122 | CONCOM08 |
| H123 | CONCOM09 |
| H124 | CONCOM10 |
| H125 | CONCOM11 |
| H126 | CONCOM12 |
| H127 | CONCOM13 |
| H128 | CONCOM14 |
| H129 | CONCOM15 |
| H130 | CONCOM16 |
| H131 | CONCOM17 |
| H132 | CONCOM18 |
| H133 | CONCOM19 |
| H134 | CONCOM20 |
| H135 | CONCOM21 |
| H136 | CONCOM22 |
| H137 | CONCOM23 |
| H138 | CONCOM24 |
| H139 | CONCOM25 |
| H140 | CONCOM26 |
| H141 | CONCOM27 |
| H142 | CONCOM28 |
| H143 | LOCAL Check |

Supplementary Table S2. Metrological data recorded at Ikenne when managed drought stress was imposed during the dry season (November-April) for eight years.

|  | Rainfall | Solar radiation | Minimum temperature | Maximum temperature | Minimum relative humidity | Maximum relative humidity |
| --- | --- | --- | --- | --- | --- | --- |
| Month | (mm) | (MJ/m²/day) | (°C) | (°C) | (%) | (%) |
| 2011/2012 | | | | | | |
| November | 21.3 | 18.7 | 22.6 | 29.4 | 78 | 94 |
| December | 0.0 | 18.6 | 19.8 | 29.7 | 66 | 92 |
| January | 1.8 | 17.5 | 20.4 | 29.7 | 64 | 94 |
| February | 47.3 | 16.0 | 23.0 | 29.7 | 79 | 92 |
| March | 93.9 | 17.4 | 23.5 | 30.1 | 68 | 93 |
| 2012/2013 | | | | | | |
| November | 109.8 | 17.5 | 23.1 | 28.9 | 85 | 94 |
| December | 0.0 | 17.8 | 21.1 | 29.3 | 68 | 94 |
| January | 0.9 | 17.7 | 21.5 | 29.8 | 66 | 94 |
| February | 66.5 | 17.5 | 22.7 | 29.8 | 64 | 93 |
| March | 116.6 | 19.3 | 24.2 | 29.8 | 87 | 93 |
| 2013/2014 | | | | | | |
| November | 24.0 | 18.1 | 23.2 | 29.1 | 87 | 93 |
| December | 10.0 | 17.3 | 21.5 | 29.6 | 68 | 93 |
| January | 52.3 | 17.2 | 22.8 | 30.3 | 81 | 95 |
| February | 0.0 | 16.2 | 23.3 | 30.5 | 71 | 91 |
| March | 56.3 | 17.7 | 23.9 | 29.7 | 81 | 93 |
| 2014/2015 | | | | | | |
| November | 94.1 | 17.3 | 23.1 | 29.3 | 83 | 95 |
| December | 12.7 | 17.8 | 21.9 | 30.5 | 65 | 89 |
| January | 22.0 | 18.5 | 18.5 | 29.4 | 59 | 89 |
| February | 20.0 | 16.4 | 23.7 | 30.2 | 82 | 93 |
| March | 49.8 | 16.6 | 24.2 | 30.1 | 77 | 93 |
| 2015/2016 | | | | | | |
| November | 135.4 | 18.7 | 23.2 | 30.2 | 82 | 93 |
| December | 0.0 | 19.2 | 17.0 | 29.3 | 61 | 89 |
| January | 25.8 | 17.9 | 19.5 | 30.7 | 56 | 88 |
| February | 0.0 | 17.5 | 22.7 | 32.1 | 63 | 87 |
| March | 131.0 | 17.2 | 24.7 | 31.2 | 81 | 92 |
| 2016/2017 | | | | | | |
| November | 23.5 | 18.2 | 23.5 | 30.3 | 81 | 92 |
| December | 8.7 | 17.6 | 22.3 | 30.2 | 68 | 91 |
| January | 15.1 | 17.7 | 21.9 | 30.5 | 63 | 91 |
| February | 13.5 | 17.6 | 23.2 | 30.7 | 64 | 92 |
| March | 188.5 | 18.9 | 24.1 | 30.6 | 85 | 92 |
| 2017/2018 | | | | | | |
| November | 24.3 | 18.2 | 22.9 | 29.4 | 79 | 94 |
| December | 18.4 | 16.5 | 21.8 | 30.0 | 74 | 93 |
| January | 1.5 | 18.7 | 19.5 | 30.0 | 67 | 85 |
| February | 87.8 | 15.7 | 23.5 | 30.5 | 70 | 92 |
| March | 108.8 | 18.6 | 23.8 | 29.9 | 85 | 93 |
| 2018/2019 | | | | | | |
| November | 30.7 | 17.7 | 23.3 | 29.8 | 78 | 94 |
| December | 0.0 | 18.0 | 20.7 | 30.1 | 69 | 88 |
| January | 35.7 | 16.9 | 22.2 | 30.6 | 71 | 90 |
| February | 98.8 | 15.7 | 23.2 | 30.2 | 65 | 94 |
| March | 64.5 | 19.0 | 24.1 | 30.1 | 82 | 93 |

Supplementary Table S3. Grain yields, *Striga* damage rating and *Striga* count of hybrids recorded in regional collaborative trials conducted under managed drought stress (MDS) and fully irrigated conditions (WW), artificial *Striga* infestation (STRIN) and non-infested conditions (STRNO) as well as in multiple rainfed field environments (MET) for eight years

|  |  |  | Grain yield (kg/ha) | | | | | Striga damage rating (1-9) | | Striga count (number) | |
| --- | --- | --- | --- | --- | --- | --- | --- | --- | --- | --- | --- |
| Hybrid | Years in trial | Hybrid group | MDS | WW | STRIN | STRNO | MET | 8 WAP | 10 WAP | 8 WAP | 10 WAP |
| H35 | **3** | DTSTR | 2477 | 5510 | 4279 | 5484 | 4340 | 3 | 4 | 46 | 53 |
| H99 | 2 | DTSTR | 2417 | 4860 | 4756 | 5446 | 4486 | 3 | 4 | 40 | 45 |
| H40 | 2 | DTSTR | 2253 | 5039 | 3304 | 4639 | 4018 | 4 | 5 | 54 | 65 |
| H34 | 2 | DTSTR | 2216 | 4272 | 3838 | 4344 | 4074 | 4 | 5 | 42 | 53 |
| H01 | **5** | DTSTR | 2173 | 4604 | 3881 | 4865 | 4209 | 4 | 5 | 53 | 64 |
| H94 | 2 | DTSTR | 2168 | 5211 | 5035 | 4418 | 4523 | 2 | 3 | 30 | 46 |
| H38 | 2 | DTSTR | 2145 | 4984 | 3254 | 4362 | 4131 | 4 | 5 | 51 | 63 |
| H70 | **4** | DTSTR | 2139 | 4869 | 3767 | 5763 | 4508 | 4 | 5 | 76 | 87 |
| H06 | **7** | DTSTR | 2123 | 4889 | 4377 | 5489 | 4307 | 3 | 4 | 45 | 52 |
| H79 | 3 | DTSTR | 2110 | 3987 | 4569 | 5477 | 4592 | 2 | 4 | 50 | 59 |
| H17 | 3 | DTSTR | 2103 | 5195 | 3472 | 4888 | 4208 | 4 | 5 | 67 | 83 |
| H86 | 3 | DTSTR | 2097 | 5144 | 4955 | 5010 | 4651 | 2 | 3 | 44 | 52 |
| H09 | **7** | DTSTR | 2069 | 4737 | 4097 | 5112 | 4302 | 3 | 5 | 43 | 50 |
| H85 | 3 | DTSTR | 2036 | 5296 | 3981 | 3975 | 4336 | 3 | 4 | 57 | 72 |
| H33 | 2 | DTSTR | 2006 | 4864 | 3417 | 4611 | 4014 | 4 | 4 | 57 | 69 |
| H78 | 3 | DTSTR | 1967 | 4865 | 4318 | 5245 | 4663 | 3 | 5 | 36 | 44 |
| H39 | 2 | DTSTR | 1956 | 5127 | 3832 | 4524 | 4145 | 3 | 4 | 43 | 51 |
| H100 | 2 | DTSTR | 1956 | 4609 | 4695 | 5641 | 4344 | 3 | 4 | 89 | 87 |
| H68 | 4 | DTSTR | 1943 | 4799 | 4341 | 5105 | 4550 | 3 | 4 | 51 | 58 |
| H80 | 3 | DTSTR | 1936 | 5112 | 3679 | 5386 | 4257 | 4 | 5 | 53 | 62 |
| H27 | 4 | DTSTR | 1931 | 4758 | 3875 | 5305 | 4080 | 3 | 4 | 46 | 64 |
| H15 | 7 | DTSTR | 1911 | 4868 | 4176 | 5154 | 4342 | 3 | 4 | 44 | 52 |
| H43 | 2 | DTSTR | 1884 | 4456 | 3280 | 4365 | 4043 | 4 | 5 | 45 | 52 |
| H11 | 5 | DTSTR | 1882 | 4168 | 4087 | 4805 | 4091 | 3 | 4 | 49 | 60 |
| H07 | 5 | DTSTR | 1868 | 4750 | 4232 | 4816 | 4388 | 3 | 4 | 43 | 49 |
| H47 | 2 | DTSTR | 1861 | 4356 | 3214 | 4703 | 3946 | 4 | 5 | 54 | 76 |
| H72 | 3 | DTSTR | 1855 | 4672 | 3798 | 5191 | 4038 | 3 | 4 | 41 | 54 |
| H83 | 3 | DTSTR | 1839 | 4967 | 4494 | 5749 | 4375 | 3 | 4 | 45 | 47 |
| H29 | 4 | DTSTR | 1826 | 4105 | 4444 | 5251 | 4272 | 3 | 4 | 43 | 48 |
| H04 | 8 | DTSTR | 1818 | 4745 | 4572 | 5284 | 4446 | 3 | 4 | 42 | 49 |
| H22 | 5 | DTSTR | 1801 | 4475 | 3529 | 4604 | 3865 | 3 | 5 | 46 | 54 |
| H10 | 4 | DTSTR | 1800 | 4755 | 3977 | 4640 | 4287 | 3 | 5 | 48 | 56 |
| H08 | 4 | DTSTR | 1794 | 4674 | 3876 | 4689 | 4009 | 3 | 4 | 45 | 52 |
| H73 | 4 | DTSTR | 1782 | 4246 | 4057 | 5172 | 4073 | 3 | 4 | 46 | 62 |
| H56 | 2 | DTSTR | 1779 | 4959 | 4294 | 5356 | 4383 | 3 | 4 | 38 | 45 |
| H16 | 7 | DTSTR | 1764 | 4988 | 4229 | 4898 | 4194 | 3 | 4 | 38 | 54 |
| H41 | 2 | DTSTR | 1758 | 4497 | 3854 | 4889 | 4105 | 3 | 4 | 41 | 49 |
| H03 | 5 | DTSTR | 1756 | 4446 | 4040 | 4827 | 4126 | 3 | 4 | 44 | 51 |
| H88 | 3 | DTSTR | 1749 | 3947 | 4595 | 4536 | 4282 | 2 | 4 | 12 | 16 |
| H21 | 6 | DTSTR | 1735 | 4534 | 4127 | 5008 | 4312 | 3 | 5 | 50 | 56 |
| H84 | 2 | DTSTR | 1731 | 4411 | 3736 | 5299 | 4711 | 3 | 5 | 43 | 62 |
| H14 | 8 | DTSTR | 1721 | 4879 | 4354 | 5107 | 4319 | 3 | 4 | 39 | 47 |
| H97 | 2 | DTSTR | 1707 | 4430 | 4217 | 4062 | 4242 | 3 | 4 | 29 | 39 |
| H13 | **8** | DTSTR | 1683 | 4440 | 4485 | 5187 | 4387 | 3 | 4 | 49 | 56 |
| H65 | 2 | DTSTR | 1641 | 4836 | 3560 | 4413 | 4341 | 3 | 4 | 39 | 47 |
| H23 | 3 | DTSTR | 1638 | 4558 | 3505 | 4654 | 3965 | 4 | 5 | 50 | 63 |
| H76 | 4 | DTSTR | 1626 | 4851 | 3922 | 5330 | 4483 | 4 | 5 | 58 | 77 |
| H75 | 4 | DTSTR | 1609 | 4710 | 4129 | 5170 | 4111 | 3 | 5 | 51 | 70 |
| H82 | 3 | DTSTR | 1600 | 4714 | 3777 | 4851 | 4555 | 4 | 5 | 41 | 52 |
| H28 | 2 | DTSTR | 1592 | 4866 | 3871 | 5089 | 4215 | 3 | 4 | 45 | 50 |
| H91 | 2 | DTSTR | 1560 | 4977 | 3009 | 4486 | 4030 | 4 | 6 | 100 | 128 |
| H05 | 4 | DTSTR | 1547 | 3393 | 3958 | 4547 | 3649 | 3 | 4 | 45 | 52 |
| H63 | 2 | DTSTR | 1540 | 4496 | 3999 | 4419 | 4041 | 3 | 4 | 34 | 40 |
| H24 | 6 | DTSTR | 1505 | 4434 | 4292 | 5181 | 4296 | 3 | 4 | 48 | 59 |
| H20 | 5 | DTSTR | 1504 | 4691 | 4331 | 5159 | 4256 | 3 | 4 | 53 | 62 |
| H89 | 2 | DTSTR | 1482 | 4211 | 3961 | 4547 | 4537 | 3 | 5 | 5 | 16 |
| H18 | 4 | DTSTR | 1468 | 4449 | 3999 | 5102 | 4310 | 3 | 5 | 46 | 57 |
| H02 | 4 | DTSTR | 1455 | 3817 | 3829 | 4177 | 4021 | 3 | 4 | 43 | 50 |
| H95 | 2 | DTSTR | 1433 | 4403 | 4604 | 5128 | 4131 | 3 | 5 | 23 | 28 |
| H55 | 2 | DTSTR | 1411 | 5066 | 4169 | 5162 | 4497 | 3 | 3 | 61 | 76 |
| H12 | 7 | DTSTR | 1409 | 4172 | 4125 | 4799 | 4163 | 3 | 4 | 38 | 45 |
| H81 | 3 | DTSTR | 1396 | 4720 | 4938 | 4895 | 4267 | 3 | 4 | 22 | 26 |
| H74 | 3 | DTSTR | 1366 | 3912 | 3993 | 5324 | 4204 | 3 | 5 | 53 | 54 |
| H19 | 6 | DTSTR | 1319 | 3819 | 3836 | 5062 | 4168 | 3 | 5 | 71 | 81 |
| H96 | 2 | DTSTR | 1310 | 3773 | 4910 | 4923 | 4441 | 3 | 4 | 28 | 28 |
| H98 | 2 | DTSTR | 1218 | 3960 | 4349 | 5223 | 4303 | 3 | 4 | 42 | 52 |
| H92 | 2 | DTSTR | 1215 | 3614 | 3509 | 4254 | 3658 | 3 | 4 | 16 | 22 |
| H87 | 3 | DTSTR | 1168 | 4358 | 4481 | 4561 | 4240 | 3 | 5 | 20 | 24 |
| H93 | 2 | DTSTR | 918 | 4264 | 4459 | 4517 | 4071 | 3 | 4 | 27 | 31 |
| H37 | 2 | DTSTR | 418 | 2538 | 2202 | 2873 | 3191 | 4 | 5 | 39 | 45 |
| H36 | 1 | DTSTR | 2511 | 6608 | 3343 | 4867 | 4056 | 4 | 6 | 52 | 69 |
| H48 | 1 | DTSTR | 2408 | 5624 | 3683 | 4299 | 4170 | 3 | 5 | 48 | 56 |
| H49 | 1 | DTSTR | 2360 | 4504 | 1678 | 4441 | 4037 | 6 | 8 | 82 | 91 |
| H109 | 1 | DTSTR | 2357 | 4578 | 5473 | 6327 | 4735 | 3 | 4 | 50 | 59 |
| H77 | 1 | DTSTR | 2311 | 4655 | 3425 | 5536 | 4279 | 4 | 6 | 73 | 47 |
| H101 | 1 | DTSTR | 2258 | 4910 | 3625 | 5404 | 4320 | 3 | 7 | 44 | 51 |
| H105 | 1 | DTSTR | 2241 | 4572 | 3891 | 5027 | 4389 | 4 | 6 | 113 | 151 |
| H69 | 1 | DTSTR | 2061 | 4213 | 3580 | 4911 | 4508 | 4 | 5 | 37 | 55 |
| H44 | 1 | DTSTR | 2031 | 4491 | 2903 | 3588 | 4111 | 3 | 5 | 35 | 42 |
| H103 | 1 | DTSTR | 2021 | 6244 | 3161 | 4525 | 4621 | 4 | 6 | 45 | 46 |
| H108 | 1 | DTSTR | 2016 | 5162 | 4652 | 5916 | 4765 | 3 | 6 | 39 | 43 |
| H42 | 1 | DTSTR | 1927 | 4898 | 3199 | 4891 | 4212 | 4 | 5 | 47 | 55 |
| H107 | 1 | DTSTR | 1902 | 4704 | 4711 | 4969 | 3896 | 3 | 4 | 53 | 57 |
| H64 | 1 | DTSTR | 1825 | 5255 | 2705 | 4378 | 3969 | 3 | 5 | 48 | 59 |
| H106 | 1 | DTSTR | 1808 | 5658 | 3718 | 5684 | 3989 | 3 | 6 | 65 | 93 |
| H51 | 1 | DTSTR | 1807 | 5442 | 3467 | 4399 | 4215 | 3 | 5 | 46 | 62 |
| H45 | 1 | DTSTR | 1798 | 6131 | 4230 | 4383 | 4060 | 3 | 4 | 47 | 57 |
| H52 | 1 | DTSTR | 1767 | 4424 | 1811 | 4322 | 3866 | 5 | 6 | 45 | 53 |
| H60 | 1 | DTSTR | 1746 | 3101 | 2095 | 2958 | 3159 | 3 | 4 | 20 | 29 |
| H104 | 1 | DTSTR | 1724 | 4752 | 4964 | 4668 | 4905 | 3 | 3 | 29 | 28 |
| H26 | 1 | DTSTR | 1665 | 4907 | 2347 | 4894 | 3997 | 5 | 7 | 109 | 128 |
| H112 | 1 | DTSTR | 1657 | 4211 | 3985 | 4554 | 4646 | 3 | 4 | 42 | 43 |
| H31 | 1 | DTSTR | 1618 | 4630 | 3324 | 4479 | 4016 | 3 | 4 | 50 | 58 |
| H90 | 1 | DTSTR | 1610 | 5052 | 5145 | 6491 | 4427 | 3 | 3 | 33 | 32 |
| H67 | 1 | DTSTR | 1580 | 4808 | 4529 | 5300 | 4408 | 3 | 5 | 9 | 16 |
| H66 | 1 | DTSTR | 1520 | 4162 | 3373 | 4170 | 3904 | 3 | 4 | 45 | 53 |
| H53 | 1 | DTSTR | 1508 | 3979 | 3902 | 4661 | 3654 | 3 | 4 | 20 | 27 |
| H110 | 1 | DTSTR | 1483 | 4413 | 5268 | 5263 | 4030 | 3 | 3 | 71 | 83 |
| H58 | 1 | DTSTR | 1472 | 4349 | 2456 | 3567 | 3682 | 3 | 5 | 36 | 43 |
| H54 | 1 | DTSTR | 1443 | 3767 | 1904 | 4087 | 3666 | 7 | 7 | 188 | 196 |
| H50 | 1 | DTSTR | 1437 | 4183 | 3044 | 4922 | 4477 | 3 | 5 | 50 | 57 |
| H71 | 1 | DTSTR | 1434 | 4837 | 4042 | 5777 | 4447 | 3 | 5 | 24 | 29 |
| H102 | 1 | DTSTR | 1351 | 4735 | 4763 | 5155 | 4002 | 2 | 4 | 34 | 35 |
| H111 | 1 | DTSTR | 1322 | 3136 | 4825 | 5330 | 3335 | 2 | 3 | 51 | 78 |
| H57 | 1 | DTSTR | 1275 | 2470 | 2027 | 2072 | 3007 | 4 | 5 | 22 | 32 |
| H25 | 1 | DTSTR | 1183 | 3211 | 2572 | 2883 | 3387 | 4 | 5 | 35 | 40 |
| H32 | 1 | DTSTR | 1130 | 2684 | 2917 | 2972 | 3086 | 4 | 5 | 41 | 45 |
| H61 | 1 | DTSTR | 1082 | 3226 | 2713 | 3094 | 2889 | 4 | 6 | 43 | 51 |
| H30 | 1 | DTSTR | 1077 | 3797 | 3177 | 4001 | 3767 | 4 | 5 | 65 | 73 |
| H62 | 1 | DTSTR | 984 | 3004 | 2574 | 3149 | 2671 | 4 | 5 | 36 | 52 |
| H46 | 1 | DTSTR | 580 | 2372 | 2819 | 3692 | 3964 | 3 | 5 | 41 | 48 |
| H59 | 1 | DTSTR | 492 | 2375 | 2838 | 2973 | 2857 | 4 | 5 | 7 | 14 |
| H114 | 8 | STRCOM | 1408 | 3870 | 3515 | 4211 | 3805 | 3 | 4 | 40 | 48 |
| H113 | 7 | STRCOM | 1230 | 3770 | 3706 | 4446 | 3867 | 3 | 4 | 35 | 39 |
| H137 | **2** | CONCOM | 2242 | 5551 | 2002 | 5032 | 4505 | 5 | 7 | 117 | 127 |
| H118 | **6** | CONCOM | 1852 | 5536 | 2328 | 4952 | 4421 | 5 | 7 | 56 | 63 |
| H131 | **2** | CONCOM | 1651 | 3492 | 1715 | 3650 | 3860 | 6 | 7 | 56 | 69 |
| H130 | **2** | CONCOM | 1605 | 3518 | 2487 | 4172 | 4096 | 4 | 6 | 47 | 56 |
| H135 | **2** | CONCOM | 1532 | 4762 | 1629 | 4712 | 4399 | 6 | 7 | 54 | 61 |
| H120 | **4** | CONCOM | 1413 | 4032 | 1980 | 4452 | 3968 | 5 | 7 | 55 | 63 |
| H138 | **2** | CONCOM | 1381 | 6086 | 1937 | 4357 | 4686 | 5 | 7 | 119 | 154 |
| H132 | **2** | CONCOM | 1336 | 3904 | 1775 | 3921 | 4298 | 5 | 7 | 57 | 65 |
| H134 | **2** | CONCOM | 1269 | 3555 | 1287 | 3033 | 3787 | 6 | 7 | 100 | 113 |
| H119 | **5** | CONCOM | 1239 | 4410 | 2280 | 4845 | 4309 | 5 | 7 | 49 | 58 |
| H117 | **5** | CONCOM | 1228 | 3191 | 1568 | 3288 | 3712 | 6 | 7 | 59 | 70 |
| H115 | **4** | CONCOM | 1217 | 4029 | 1909 | 3706 | 3904 | 5 | 7 | 88 | 106 |
| H136 | **2** | CONCOM | 1212 | 5181 | 1279 | 5739 | 4747 | 6 | 7 | 146 | 132 |
| H121 | **3** | CONCOM | 1173 | 3641 | 1389 | 3748 | 3941 | 6 | 8 | 85 | 97 |
| H127 | **4** | CONCOM | 1162 | 3372 | 2226 | 3828 | 3629 | 5 | 6 | 73 | 83 |
| H116 | 3 | CONCOM | 577 | 3169 | 2176 | 3329 | 3215 | 5 | 7 | 65 | 79 |
| H141 | 1 | CONCOM | 2886 | 6584 | 5049 | 7553 | 5529 | 4 | 7 | 29 | 30 |
| H142 | 1 | CONCOM | 2424 | 5417 | 3434 | 6528 | 5792 | 4 | 6 | 77 | 105 |
| H122 | 1 | CONCOM | 2189 | 5159 | 1672 | 4534 | 4572 | 6 | 7 | 113 | 123 |
| H125 | 1 | CONCOM | 1645 | 4320 | 2231 | 4644 | 4324 | 7 | 8 | 82 | 90 |
| H123 | 1 | CONCOM | 1623 | 3784 | 1136 | 2204 | 2898 | 5 | 8 | 56 | 66 |
| H140 | 1 | CONCOM | 1487 | 4574 | 2385 | 5476 | 4728 | 6 | 9 | 93 | 120 |
| H128 | 1 | CONCOM | 1237 | 3343 | 1670 | 4639 | 3654 | 6 | 8 | 113 | 133 |
| H129 | 1 | CONCOM | 1202 | 2843 | 2737 | 4100 | 4054 | 4 | 6 | 48 | 60 |
| H139 | 1 | CONCOM | 1022 | 5126 | 2764 | 4391 | 4524 | 4 | 8 | 113 | 125 |
| H126 | 1 | CONCOM | 922 | 3645 | 1990 | 3705 | 4044 | 5 | 7 | 53 | 61 |
| H124 | 1 | CONCOM | 744 | 3884 | 3384 | 5499 | 4592 | 4 | 7 | 56 | 65 |
| H133 | 1 | CONCOM | 449 | 2974 | 1348 | 2686 | 2747 | 4 | 6 | 42 | 50 |
| H143 | 8 | LOCAL | 793 | 2235 | 1752 | 3243 | 3550 | 5 | 7 | 61 | 79 |
| Mean |  |  | 1643 | 4422 | 3473 | 4658 | 4132 | 4 | 5 | 52 | 62 |
| LSD (0.05) |  |  | 863 | 1425 | 1262 | 1337 | 657 | 1 | 1 | 37 | 43 |
| Repeatability |  |  | 0.52 | 0.55 | 0.80 | 0.56 | 0.54 | 0.86 | 0.85 | 0.66 | 0.65 |

8 WAP = 8 weeks after planting, 10 WAP = 10 weeks after planting

Supplementary Table S4. Genetic correlation between grain yield and other traits measured each under manged drought stress and artificial Striga infestation

| Trait combinations | Genotypic correlations under managed drought stress (MDS) |
| --- | --- |
| Yield with anthesis date | -0.36±0.07 |
| Yield with anthesis-silking interval | -0.63±0.14 |
| Yield with plant height | 0.35±0.13 |
| Yield with ear height | 0.35±0.13 |
| Yield with ears per plant | 0.81±0.05 |
| Trait combinations | Genotypic correlations under *Striga* infestation (STRIN) |
| Yield with silking days | -0.41±0.06 |
| Yield with anthesis days | -0.37±0.06 |
| Yield with plant height | 0.30±0.08 |
| Yield with Striga damage rating at 8 WAP | -0.87±0.03 |
| Yield with Striga damage rating at 10 WAP | -0.93±0.02 |
| Yield with Striga count at 8 WAP | -0.78±0.09 |
| Yield with Striga count at 10 WAP | -0.63±0.08 |
| Yield with ear aspect scores | -0.92±0.02 |
| Yield with ears per plant | 0.92±0.02 |

Supplementary Table S5. Correlations of actual values of traits measured under manged drought stress and artificial Striga infestation with their corresponding first two principal component axes scores

| Traits | PC1 | PC2 |
| --- | --- | --- |
|  | Managed drought stress (MDS) | |
| Anthesis date | -0.41**** | -46**** |
| Anthesis-silking interval | 0.02 | 0.49**** |
| Plant height | 0.88***** | 0.63**** |
| Ear height | 0.89**** | 0.63**** |
| Ears per plant | 0.54**** | -0.32*** |
| Variance | 0.41 | 0.24 |
|  | Artificial *Striga* infestation (STRIN) | |
| Silking days | 0.45**** | 0.84**** |
| Anthesis days | 0.21* | 0.94**** |
| Plant height | -0.57**** | 0.29*** |
| Striga damage rating at 8 WAP | 0.93**** | -0.11 |
| Striga damage rating at 10 WAP | 0.90**** | -0.07 |
| Striga count at 8 WAP | 0.79**** | -0.13 |
| Striga count at 10 WAP | 0.78**** | -0.14 |
| Ear aspect scores | 0.88**** | 0.00 |
| Ears per plant | -0.86**** | 0.04 |
| Variance | 0.55 | 0.20 |
