# Supplementary material for: Estimating genetic gains for tolerance to stress combinations in tropical maize hybrids
Source: Front Genet. 2022 Dec 8;13:1023318. doi: 10.3389/fgene.2022.1023318 (PMC9779929; doi:10.3389/fgene.2022.1023318)
Supplement: Supplementary file 2 [file Table2.DOCX]

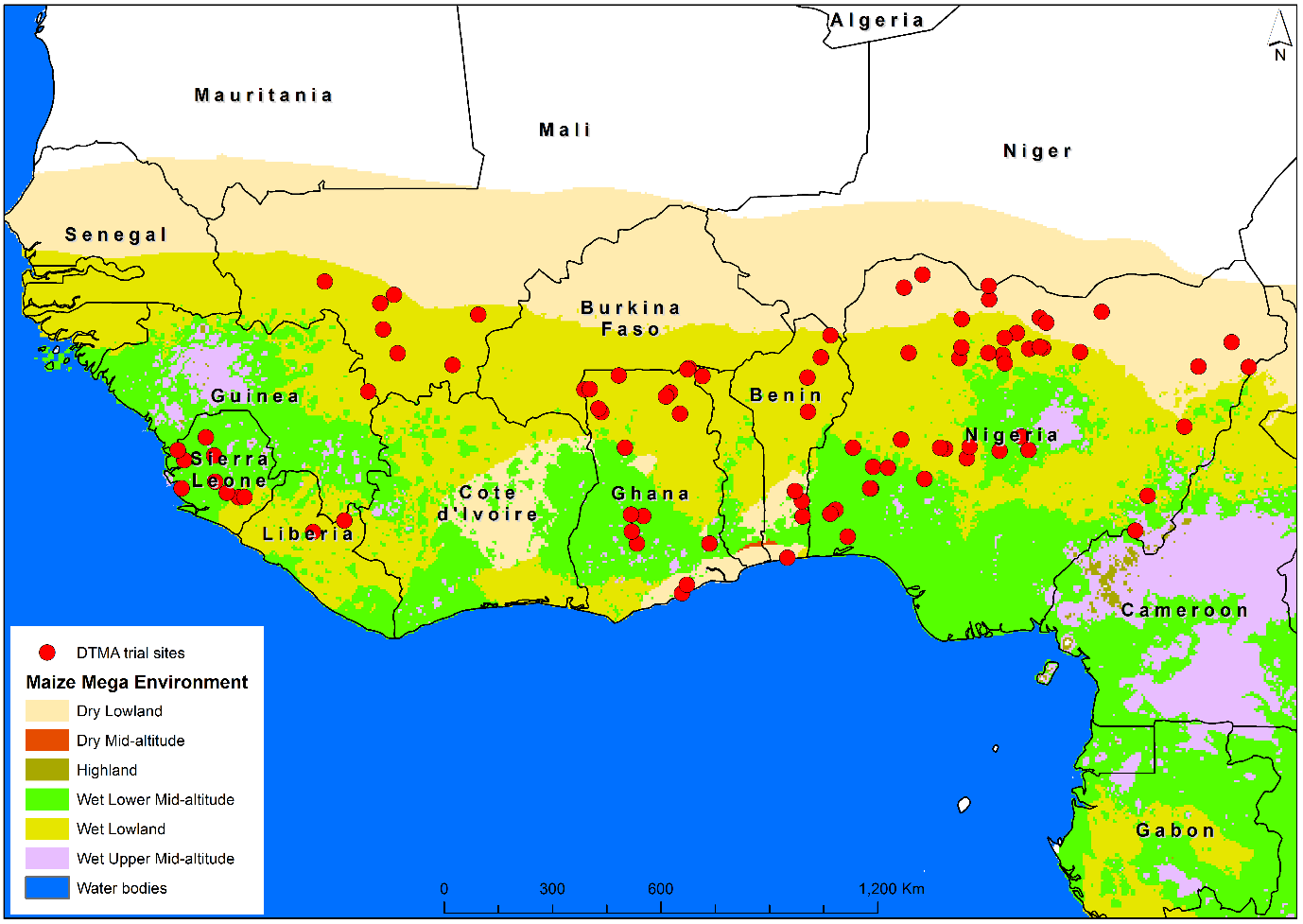


Supplementary Figure S1. Testing sites used for running the regional trials from 2012 to 2019.


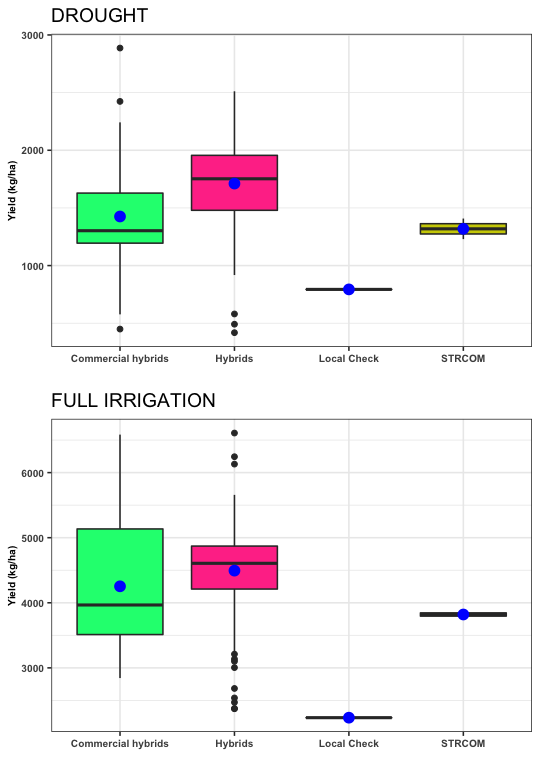


Supplementary Figure S2. Grain yields of commercial hybrids (CONCOM), Hybrids (DTSTR), *Striga* resistant (STRCOM) commercial hybrids and the local check recorded in regional collaborative trails evaluated for eight years under managed drought stress and full irrigation.


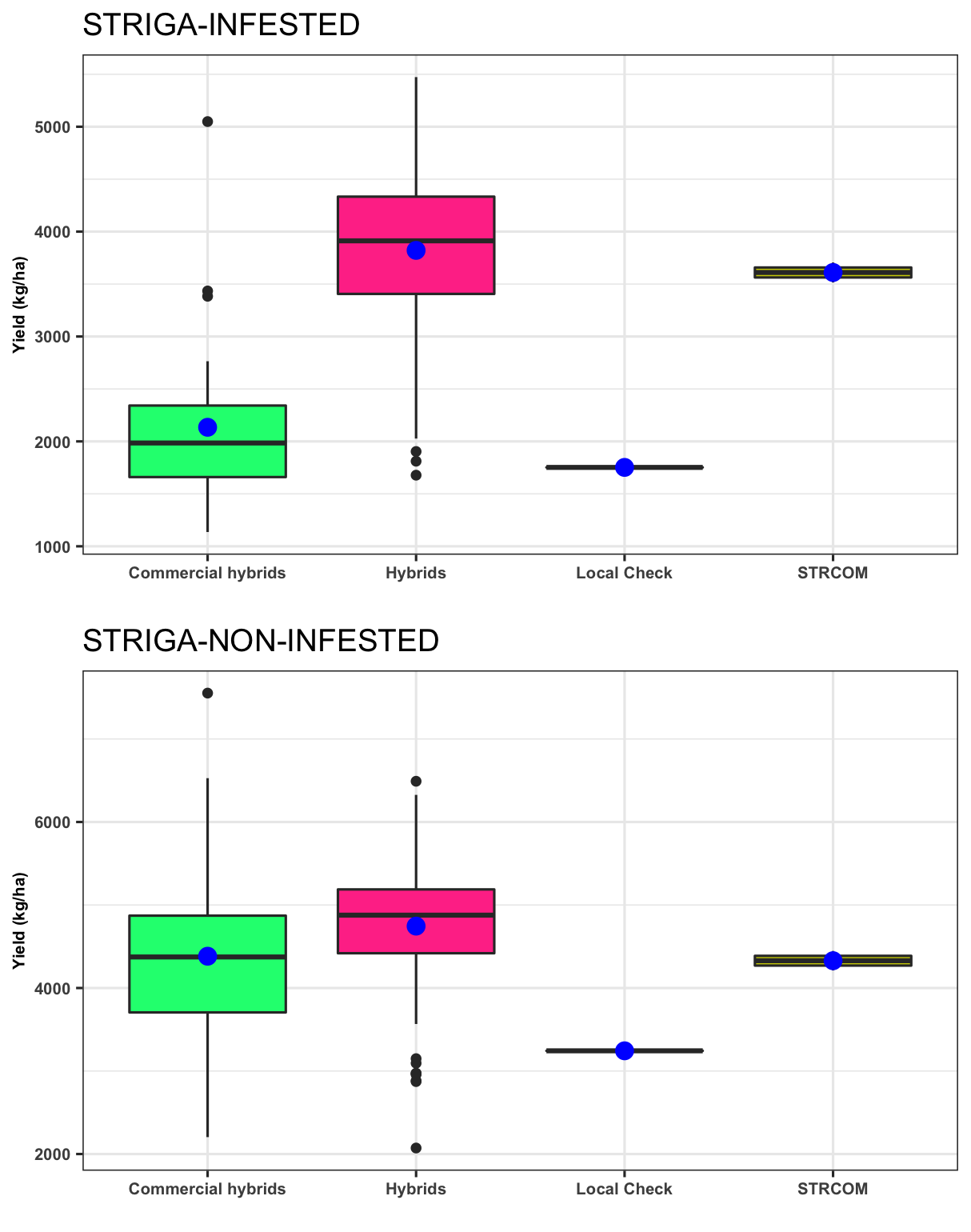


Supplementary Figure S3. Grain yields of commercial hybrids (CONCOM), Hybrids (DTSTR), *Striga* resistant (STRCOM) commercial hybrids and the local check recorded in regional collaborative trails conducted at two locations for eight years under artificial *Striga* infested and non-infested conditions.


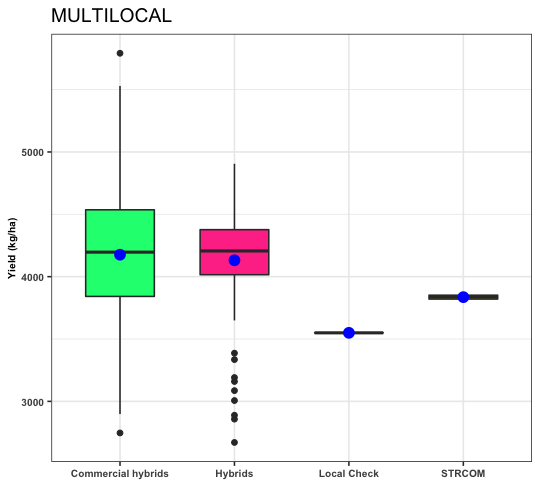


Supplementary Figure S4. Grain yields of commercial hybrids (CONCOM), Hybrids (DTSTR), *Striga* resistant (STRCOM) commercial hybrids and the local check recorded in regional collaborative trails conducted for eight years across multiple test locations in West Africa.
